# Sleepyhead, deadly awakening: the dynamics of metastatic organotropism, tumor dormancy and therapeutic implications

**DOI:** 10.3389/fonc.2025.1701031

**Published:** 2025-12-01

**Authors:** Soumyajit Sarkar, Suresh P. K.

**Affiliations:** Department of Bio-Medical Sciences, School of Bio Sciences and Technology, Vellore Institute of Technology (VIT), Vellore, Tamil Nadu, India

**Keywords:** tumor dormancy, cancer recurrence, organotropism, microenvironmental signaling, therapeutic approaches

## Abstract

As per the global mortality-related data, metastasis and tumor-related relapse are the major determinants of cancer-related deaths. This phenomenon is largely driven by tumor dormancy - a state in which disseminated tumor cells (DTCs) persist in a non-proliferative phase. These dormant cells evade immune surveillance and resist conventional therapies, contributing to late relapse and metastatic outgrowth. Dormancy is maintained through intricate crosstalk between cancer cells and the microenvironment, involving extracellular matrix components, and various cellular signaling pathways. However, changes in these microenvironmental cues can disrupt this balance and reactivate dormant cells, leading to their proliferation and metastatic colonization. The undetectability of dormant DTCs complicate therapeutic targeting, underscoring the need to elucidate the molecular and epigenetic mechanisms that regulate dormancy maintenance and escape. This review explores the key signaling mechanisms and microenvironmental influences that regulates the tumor dormancy. Furthermore, we discuss emerging therapeutic strategies aimed at eradicating dormant cancer cells - either by maintaining dormant state, reactivating and sensitizing dormant cells to chemotherapy, or directly eliminating dormant populations. A deeper understanding of dormancy biology holds promise for developing innovative interventions to prevent recurrence and improve long-term patient survival.

## 1 Introduction

Cancer metastasis and relapse stands as one of the leading cause of mortality globally (1, 2). The primary approach to cancer treatment is surgical removal of tumors, typically accompanied by chemoradiotherapy to eliminate cancer cells that cannot be surgically accessed. However, even after patients are deemed clinically cancer-free post-treatment, relapse with distant metastasis is common. These metastatic cells often develop resistance to chemoradiation, enabling them to persist and spread, forming new metastatic colonies that compromise the function of vital organs. This phenomenon is known as minimal residual disease (MRD) (3).

During the multistep process of metastasis including local invasion, intravasation, extravasation, and colonization, a small subset of disseminated tumor cells (DTCs) may transition into a state of cell-cycle arrest, where the cells remain viable but do not proliferate. Eventually, in response to signals from their new microenvironment at the secondary site, these cells may reacquire proliferative capacity and undergo adaptive changes to integrate with their surroundings. It leads to metastatic outgrowth and the development of secondary tumors. This latency-associated behavior, collectively referred to as cancer dormancy, has gained significant attention as a major contributor to delayed metastatic relapse and resistance to therapy. The concept of dormancy was first introduced in 1934 by Rupert Willis, who observed delayed metastases in patients without signs of local recurrence and suggested that the cancer cells had entered secondary tissues (4). Later, in 1954, Geoffrey Hadfield proposed that such late recurrences were caused by cells that had undergone a “temporary mitotic arrest” (5). The terminology used to describe cancer cell dormancy remains inconsistent, with a range of overlapping terms complicating the field. Cells referred to as “quiescent”, “drug-tolerant persisters”, “slow-cycling”, “senescent”, or “cancer stem cell-like” are frequently grouped under the broad category of “dormant” cancer cells (6). Dormancy encompasses cellular dormancy, where individual DTCs remain in a quiescent and/or senescent state, and tumor mass dormancy, in which small metastatic lesions are held in check by extrinsic factors such as immune surveillance or insufficient angiogenesis.

An increasing volume of research has shed light on the molecular pathways that regulate cellular dormancy and subsequent reactivation. A key aspect of these processes is the dynamic interplay between tumor cells and their microenvironment. Cancer cells can reversibly change their signaling, switching between proliferative and dormant states following primary treatment. This phenotypic plasticity, driven by epigenetic changes, transcriptional regulation, and tumor heterogeneity, presents significant challenges in metastasis research (7). Despite the identification of various intrinsic and extrinsic factors that regulate dormancy and reactivation, developing effective clinical therapies that target dormant cancer cells remains a pressing need. This article aims to offer insights into the mechanisms of cancer cell dormancy and reactivation, their roles in cancer progression, and potential therapeutic strategies. These strategies include maintaining dormancy to prevent tumor progression, reawakening dormant cells for targeted destruction, and directly eliminating dormant cells. Each approach is focused on developing novel anticancer agents that specifically target the dormant state, aiming to reduce relapse and improve long-term treatment outcomes.

## 2 Quiescence vs. senescence and beyond

**Figure 1:**
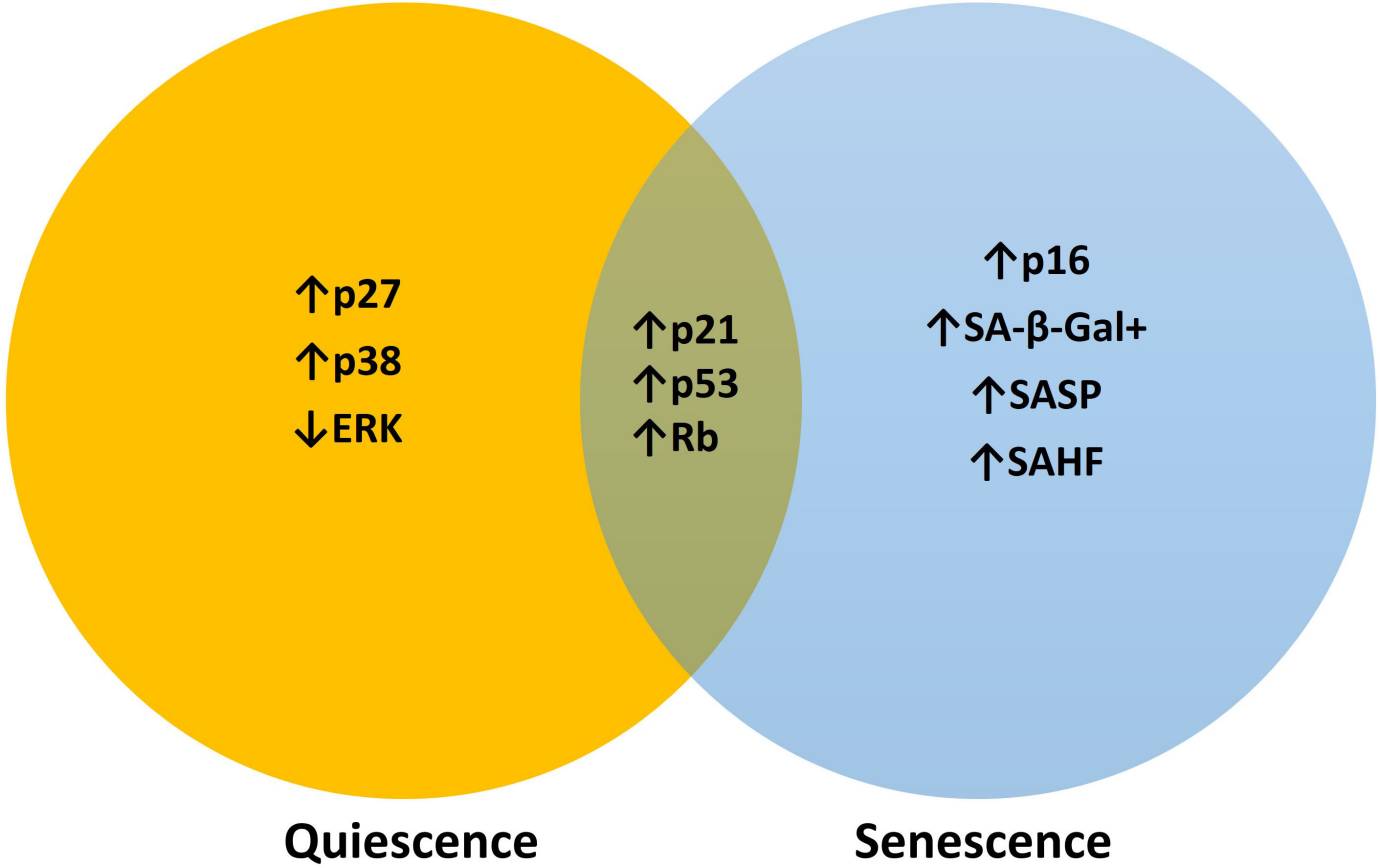
The common molecular and phenotypic features between quiescence and senescence suggest that these states lie on a spectrum rather than being entirely distinct. Key molecular players that are distinct or overlapping with each other are highlighted.

While both quiescence and senescence involve a cessation of cellular proliferation, they differ markedly in their regulatory mechanisms, functional outcomes, and implications for disease progression. Quiescence is a reversible and precisely controlled growth arrest typically occurring in the G_0_ to G_1_ phase that enables cells to rapidly re-enter the cell cycle in response to favorable environmental signals, thus, quiescence may be the primary mechanism that characterizes cellular dormancy (8). In contrast, senescence constitutes a more stable, often irreversible, growth arrest in G_1_-G_1_/S cell cycle check points typically induced by cellular stress or genotoxic damage (9). Senescent cells exhibit distinct morphological changes and acquire a characteristic senescence-associated secretory phenotype (SASP), which comprises a complex mix of cytokines, chemokines, proteases, and growth factors (10). This secretory profile exhibits a dual function within the tumor microenvironment (TME). On one hand, it can contribute to tumor suppression by enforcing cell cycle arrest and facilitating immune-mediated clearance of tumor cells. On the other hand, it can induce tumor progression and metastatic colonization by remodeling the extracellular matrix (ECM), enhancing angiogenesis, and fostering a pro-inflammatory microenvironment that supports tumor growth and immune evasion (11). Senescence is traditionally characterized as a state of permanent cell cycle arrest, however, emerging evidence indicates that senescence may be reversible under specific conditions, and it may still contribute to delayed tumor relapse (12). Dormant tumor cells are inherently quiescent, but quiescence is not a uniform state. It represents a spectrum of cellular behaviors shaped by diverse triggers and microenvironmental cues that can either maintain growth arrest or prepare cells for reactivation. Although quiescence and senescence differ in stability and outcome, they share overlapping mechanisms and may exist along a continuum rather than as distinct states. These two cell types share some molecular regulators (**Figure 1**), such as cyclin-dependent kinase inhibitors (CDKis) and the retinoblastoma (Rb)/E2F pathway, and they may have overlapping gene expression patterns that help them survive and evade the immune system. Understanding these shared and distinct molecular markers are essential for an improved understanding of their relative contribution to the dormancy state (induction and dormancy). as well as in the development of therapeutic strategies aimed at targeting dormant tumor populations (6).

Beyond quiescence and senescence, tumor dormancy is reinforced by distinct but interrelated subpopulations - slow-cycling cells (SCCs), drug-tolerant persisters (DTPs), and cancer stem cells (CSCs)-each employing unique adaptive strategies to withstand environmental and therapeutic stress. Slow-cycling cells (SCCs) exhibit extended cell-cycle durations and reversible arrest in the G_0_/G_1_ phase. These cells typically express high levels of cyclin-dependent kinase inhibitors p21^Cip1/Waf1^ and p27^Kip1^, display reduced Ki-67 expression, and maintain hypo-phosphorylated retinoblastoma (Rb) protein. Activation of stress-response mechanisms, such as phosphorylated eIF2α, elevated p53, and enhanced autophagy through ATG5 and ATG7, supports their survival under unfavorable conditions. Transcriptional and epigenetic regulators including JARID1B (KDM5B) and FOXO1 contribute to chromatin remodeling and maintenance of this dormant phenotype. Importantly, these cells evade chemotherapeutic elimination and can re-enter the proliferative cycle once stress is relieved, acting as a reservoir for tumor recurrence (13, 14).

Drug-tolerant persister (DTP) cells arise through reversible, non-genetic reprogramming rather than fixed mutations. They exhibit a transcriptionally repressed, quiescent-like state driven by chromatin-modifying enzymes such as KDM5A and EZH2, which remodel histone methylation patterns. DTPs sustain survival by engaging bypass signaling networks -including IGF-1R, AXL and Wnt/β-catenin, while enforcing cell-cycle arrest via p21 and p27 upregulation. These cells originate from pre-existing phenotypic heterogeneity, and their transient drug-tolerant state can be inherited across a few generations. Upon withdrawal of therapeutic pressure, DTPs resume proliferation, though persistent stress exposure may select for stable resistance through secondary mutations (15).

Cancer stem cells (CSCs) are functionally defined by their ability to self-renew, differentiate into diverse tumor lineages, and initiate tumor growth (16). Their marker profile varies by cancer type -CD44^high^/CD24^low^/ALDH1^high^ in breast, CD133^+^ in brain, LGR5^+^ in colorectal, and CD34^+^/CD38^−^ in hematologic malignancies. CSC maintenance depends on developmental signaling pathways such as Notch, Wnt/β-catenin, JAK/STAT3, and Hedgehog (17), while transcription factors SOX2, OCT4 NANOG, and epigenetic regulators BMI1 uphold pluripotency and stemness. Their resistance to therapy stems from quiescence, overexpression of ATP-binding cassette (ABC) transporters, efficient DNA repair systems, and anti-apoptotic signaling. Moreover, CSCs exhibit remarkable plasticity, often undergoing epithelial-to-mesenchymal transition (EMT) or dedifferentiation from non-stem cancer cells under therapeutic stress (18, 19).

Substantial overlap exists among these cellular states. Many CSCs reside in a slow-cycling phase, DTPs acquire stem-like characteristics, and SCCs often express canonical CSC markers. Collectively, these populations form a dormant, drug-resistant reservoir that sustains MRD and underlies metastatic relapse after therapy.

## 3 Spatio-temporal regulation of tumor dormancy during metastatic progression

Metastasis represents a complex, multistep cascade in which cancer cells detach from the primary tumor, enter and survive within the circulatory system, and ultimately establish secondary growths in distant organs. During this journey, DTCs undergo alternating phases of proliferation and dormancy, governed by evolving molecular and microenvironmental cues.

Two principal mechanistic models have been proposed to explain tumor cell dissemination: the linear (late dissemination) and the parallel progression (early dissemination) models (20). The linear model posits that tumor cells undergo successive rounds of natural selection within the primary tumor, ultimately allowing the most adaptable and aggressive clones to disseminate and establish metastases in distant organs. In contrast, the parallel progression model suggests that tumor cells can begin to disseminate at the initial phases of tumorigenesis, sometimes even before the primary tumor becomes clinically detectable (20, 21). The concept that malignant cells can remain undetected for extended periods is not restricted to DTCs but also extends to occult primary tumors that exhibit dormancy-like features. These small, clinically silent lesions, typically less than 5 mm in size, are often identified incidentally during autopsies of individuals who had no prior cancer diagnosis (22). Although the concept of early dissemination has recently gained increasing acceptance, the linear model cannot be entirely disregarded. It is likely that the timing of dissemination depends on multiple tumor-intrinsic and microenvironmental factors, representing a continuum that spans from early to late events across different patient contexts. Nevertheless, the precise determinants governing the onset of dissemination and the subsequent outgrowth of DTCs remain incompletely understood (21).

**Figure 2:**
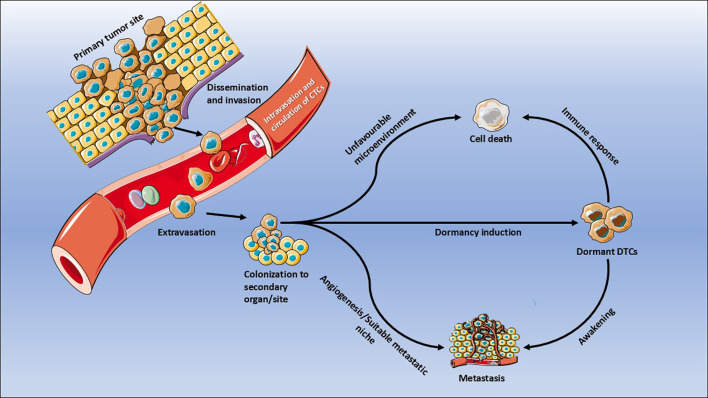
Key steps in the metastatic cascade and the induction of tumor dormancy. The schematic outlines critical phases of metastatic progression, beginning with local invasion and dissemination from the primary tumor, followed by intravasation into the bloodstream and circulation as circulating tumor cells (CTCs). Upon reaching distant organs, CTCs undergo extravasation and attempt colonization. Depending on the microenvironment, CTCs may either undergo cell death due to unfavorable conditions, or survive and proliferate if a supportive metastatic niche and angiogenic signals are present. Alternatively, exposure to dormancy-inducing cues may result in the formation of dormant disseminated tumor cells (dDTCs), which can persist in a quiescent and/or a senescent state. These dDTCs may later be eliminated by immune surveillance or reawaken to initiate metastatic outgrowth under permissive conditions.

Among these determinants, the acquisition of migratory and invasive potential through EMT plays a pivotal role. During EMT, a subset of tumor cells downregulates epithelial markers such as E-cadherin, leading to weakened cell-cell adhesion and enhanced motility, thereby facilitating intravasation and early dissemination (23). However, many retain partial epithelial traits, resulting in a hybrid epithelial-mesenchymal phenotype (24) that facilitates collective invasion while preserving essential survival signaling. Once detached, cancer cells degrade the ECM via matrix metalloproteinases (MMPs) (25) and intravasate into the bloodstream as CTCs. CTCs can exist as single cells or multicellular clusters; the latter retain epithelial junctions (E-cadherin, EpCAM), which confer apoptosis resistance and greater metastatic efficiency (26, 27). The circulatory phase imposes severe mechanical, immune, and metabolic stress. These stresses eliminate most CTCs, leaving only a minor subpopulation capable of surviving transit and extravasating into distant tissues. Upon reaching the secondary site, DTCs encounter foreign organ microenvironments that determine their fate-death, dormancy, or proliferation (21) (**Figure 2**). These secondary niches often lack the mitogenic and survival cues present in the primary tumor, compelling DTCs to enter a reversible G_0_-G_1_ cell cycle arrest. This state is marked by reduced expression of proliferation markers such as Ki-67 and PCNA, indicative of a dormant phenotype (28, 29). Dormancy at this stage is maintained through soluble mediators such as TGFβ2, BMPs, and thrombospondin-1 (TSP-1), which suppress mitogenic pathways and sustain growth arrest (30–32). The TME thus becomes a decisive regulator of DTC fate. Stromal elements, including fibroblasts, adipocytes, endothelial, and immune cells- not only secrete dormancy-inducing factors but also modulate the metabolic substrate landscape. Cancer-associated fibroblasts (CAFs), a subtype of stromal population, remodel the ECM and enhance collagen deposition (33), generating mechanical stiffness associated with dormancy induction in oral and breast cancers (34–36).

Collectively, these findings emphasize that dormancy represents a reversible, plastic state under continuous regulation by angiogenic balance, immune surveillance, and metabolic adaptation. The rate of inter-convertibility between dormant and proliferative phases depends on the niche microenvironment, immune surveillance, stromal composition, and metabolic flexibility.

### 3.1 Role of pre-niche and organotropism in secondary cancer metastasis and dormancy

The concept of site-specific metastasis has been a subject of scientific exploration since 1889, when the English surgeon Stephen Paget proposed the famous “seed and soil” theory (37). In this analogy, the metastasizing cancer cells are likened to “seeds”, while the microenvironment of organs that favor metastasis are seen as the “soil”. Paget’s theory suggested that the spread of cancer was not random but rather dependent on a suitable interaction between the disseminated cancer cells and the microenvironment of the secondary site.

The “seed and soil” hypothesis alone does not fully explain the selective organotropism of metastasis observed in different tumor subtypes. In a study using a chick embryo model, MDA231-BoM cells formed bone-specific metastases; MDA231-BrM2 cells preferentially metastasized to the brain; MDA231-LM2 cells showed exclusive tumor burden in the lungs; while the parental MDA231 cell line predominantly produced metastatic lesions in the liver (38). Modern research, leveraging advanced high-throughput technologies, has further illuminated the complexity of this relationship. It is now understood that the interaction between the “seeds” (DTCs) and the “soil” (target organ microenvironment) is not one-sided but involves dynamic and bidirectional communication. For instance, extracellular vesicles (EVs) released by cancer cells can be taken up by host cells, such as resident macrophages in distant organs. These EVs can trigger responses in the target organ, including profibrotic and proinflammatory changes, which subsequently prepare the environment to support future metastasis (39).

Early research indicated the role of chemokines in guiding cancer cells to specific organs. For instance, lymph nodes and lungs produce chemokines like CXCL12 and CCL21, which interact with their corresponding receptors, CXCR4 and CCR7, found on breast cancer cells (40). This chemokine-receptor binding helps steer the cancer cells toward these organs, promoting site-specific metastasis. However, more recent research on exosomes suggests that integrins on their surface play a more significant role in directing metastatic spread (41). Beyond carrying factors involved in pre-metastatic niche formation, tumor-derived exosomes possess specific integrins that influence their retention and uptake in target organs. Exosomes enriched with ITGα6β4 or ITGα6β1 tend to accumulate in the lungs, those with ITGαvβ5 in the liver, and those containing ITGβ3 in the brain (39), showing the metastatic preferences of the tumor cells they originate from.

The lungs, brain, bone, and liver represent the most common sites of site-specific metastasis. These patterns are influenced by factors such as microenvironmental components and secreted factors, as outlined in **Table 1**.

**Table 1:** Key factors contributing to the organotropism of cancer.

Target organs (Secondary site)	Primary tumor site	Category	Factors	Mechanism of action	References
Lung	Melanoma	ECM glycoprotein, adhesion molecule	SPARC, VCAM1	SPARC facilitates lung metastasis by enhancing vascular permeability and promoting extravasation via endothelial VCAM1 dependent manner.	(42)
	Breast cancer	Secreted glycoprotein	ANGPTL4	In breast cancer, ANGPTL4 expression correlates to tumor cell extravasation.	(43)
	Breast cancer	ECM protein	Tenascin C	An ECM protein from breast cancer cells, activates Notch and Wnt signaling in lung niches to initiate metastasis.	(44)
	Melanoma	Exosome	Tumor-derived extracellular vesicles (TEV)	EVs derived from melanoma cells downregulate IFNAR1 and CH25H in normal lung cells, thereby promoting a microenvironment conducive to lung metastasis	(45)
Brain	Breast cancer	Enzyme	Cathepsin S	Cathepsin S degrades junctional adhesion molecule JAM-B, aiding their transmigration across the blood–brain barrier (BBB).	(46)
	Breast cancer	miRNA/Extracellular vesicle	miR-181c (EV-associated)	miR-181c in EVs targets the PDPK1/cofilin pathway, disrupts cytoskeletal actin dynamics, mislocalizes tight junction proteins, reduces transendothelial electrical resistance (TEER), increases BBB permeability, thereby facilitates metastasis.	(47)
	Breast cancer	Glycosyltransferase	a2,6-sialyltransferase ST6GALNAC5	Mediates adhesion and penetration across BBB	(48, 49)
	Breast cancer	Enzyme	Heparanase (HPSE)	Promotes transendothelial migration through ECM degradation	(50)
	Breast cancer	Chemokine	CCL2	Induced by PTEN loss, recruits pro-metastatic myeloid cells	(51)
	Breast and lung cancer	Adhesion molecule/Gap junction component	PCDH7 + CX43	Forms tumor–astrocyte gap junctions to transfer cGAMP, activating the STING pathway and inducing interferon-α (IFNα) and tumor necrosis factor (TNF) production. These cytokines, through paracrine signaling, activate STAT1 and NF-κB pathways, thereby promoting tumor cell proliferation.	(52)
	Lung cancer	Astrocyte-secreted cytokines	TNF-α, IL-1β, and IL-6	Astrocyte-secreted cytokines promote tumor proliferation in the brain microenvironment, demonstrated using an intracardiac inoculation model of human lung cancer-derived (HARA-B) cells in nude mice to establish experimental brain metastases.	(53)
Bone	Prostate cancer	Tumor Subtype Specific gene fusion	TMPRSS2-ERG fusion	TMPRSS2-ERG fusion increases osteomimicry and osteoblastic lesion formation, thereby supporting osteoblastic metastasis.	(54, 55)
	Lung cancer	Tumor Cell Surface Markers	CD24, Discoidin domain receptor-1 (DDR1),	These markers support adhesion, invasion, colonization.	(56, 57)
	Breast and prostate cancer	Stromal/Secretory factor	Osteopontin	Activates PI3K/Akt/mTOR pathway to promote tumor cell proliferation, migration, and invasion; upregulates GPX4 to protect tumor cells from lipid peroxidation and ferroptosis, enhancing survival and metastatic potential	(58)
	Breast cancer	Stromal/Secretory factor	RANKL	Promotes migration, invasion, and metastasis through NF-κB pathway	(59, 60)
	Breast cancer	Immune-mediated	CD3+ T cells, CD19+ B cells	Secreted by tumor-primed T and B cells; induces osteoclastogenesis and bone resorption, creating a niche for tumor colonization.	(61)
	Breast cancer	Immune-mediated	Prostaglandin E2	Recruits Tregs (CD4^+^CD25^+^) to bone metastasis site, promoting immune evasion and niche formation *in vivo.*	(62)
Liver	Colorectal cancer	ECM Remodeling	PAD4	Citrullination of ECM promotes EMT and liver colonization *in vivo.*	(63)
	Breast cancer	Tumor -Hepatocyte Interaction	Claudin-2	Mediates direct interaction with hepatocytes and enhances liver colonization.	(64, 65)
	Pancreatic cancer	Exosome	Macrophage migration inhibitory factor (MIF)-enriched exosomes, TGF-β, fibronectin	Exosomes derived from pancreatic ductal adenocarcinoma (PDAC) cells, enriched with MIF, activate Kupffer cells and induce the release of TGF-β. This cytokine, in turn, stimulates hepatic stellate cells to secrete fibronectin, thereby facilitating metastatic progression, as demonstrated through combined human and mouse *in vivo* studies.	(66)
	Pancreatic cancer	Secreted signaling factor	Metastasis-associated macrophages (MAMs)	MAM-derived granulin activates hepatic stellate cells, inducing a fibrotic microenvironment that supports metastasis, demonstrated through combined human and mouse studies.	(67)

### 3.2 Metabolic plasticity in tumor dormancy

For decades, tumor metabolism was largely understood through the concept of the “Warburg effect”, where cancer cells predominantly rely on aerobic glycolysis, metabolizing glucose into lactate despite the availability of adequate oxygen (68, 69). However, this simplified view has evolved. It is now clear that cancer metabolism is highly heterogeneous, and aerobic glycolysis is not a universal feature of all tumors (23). Metabolic diversity arises from intrinsic factors, such as genetic mutations and differentiation status, as well as extrinsic influences like oxygen and nutrient availability and interactions with stromal and ECM components (70). Within this complex landscape, oxidative phosphorylation (OXPHOS) and alternative energy pathways have gained recognition as major contributors to the metastatic adaptation (23).

Tumors contain subpopulations of proliferative, slow-cycling/dormant, and apoptotic cells, each sustained by distinct metabolic programs. Slow-cycling and/or dormant cancer cells tend to depend on mitochondrial respiration rather than glycolysis for their survival. This reliance on OXPHOS is now recognized as a metabolic signature of DTC dormancy (71). This is demonstrated by the findings of Roesch et al. (72), who reported an enrichment of slow-cycling melanoma cells expressing the H3K4-demethylase JARID1B. These cells exhibit elevated mitochondrial metabolism, and targeting mitochondrial respiration sensitizes them to therapy. Inhibition of key cell-cycle regulators, such as CDK4/6 and CDK2, not only promotes quiescence but also alters cellular energy production. In melanoma, CDK4/6 inhibition enhances glutamine utilization and fatty acid oxidation (FAO) to sustain mitochondrial respiration (73). Similarly, in a gastric cancer model, suppression of CDK2 activity promotes OXPHOS through SIRT5 while reducing glycolysis (74). Dormant breast cancer (75) and pancreatic cancer (76) cells also rely on mitochondrial energy metabolism, reflecting a broader shift from glycolytic, anabolic growth to catabolic, energy-efficient pathways that maintain redox balance and long-term viability.

Adenosine monophosphate-activated protein kinase (AMPK) acts as a central regulator of this metabolic adaptation. By sensing cellular energy status (77), AMPK promotes catabolic pathways, including autophagy via ULK1 activation, fatty acid oxidation through inhibition of acetyl-CoA carboxylase, and mitochondrial biogenesis via PGC-1α (78–80). In dormant estrogen receptor-positive (ER+) breast cancer cells, AMPK signaling supports survival through enhanced FAO-driven OXPHOS (81). Dormant cells also exhibit metabolic flexibility, switching between substrates to sustain mitochondrial function when glucose availability is limited. For example, a subset of CD44^bright^ cells in an oral carcinoma model display high expression of the fatty acid translocase CD36 and rely on lipid uptake and β-oxidation for metastasis formation (82).

CSCs exhibit remarkable metabolic plasticity during dormancy, Lower levels of reactive oxygen species (ROS) or enhanced glutathione (GSH) are closely associated with the quiescent state of CSCs and contribute to chemoresistance (83). It helps to maintain the reduced intracellular environment necessary for dormancy. CSCs isolated from diverse tumor types often display a stronger reliance on glycolysis compared to their non-stem counterparts, as evidenced by elevated expression of glycolytic genes and a concomitant reduction in OXPHOS-associated transcripts. For instance, ALDH^+^ breast CSCs derived from MDA-MB-231 and MCF-7 mammospheres exhibit higher expression of PDK1 and lower levels of PDH relative to non-CSCs. Notably, silencing PDK1 in MDA-MB-231 cells reduces stemness-associated properties (84). However, a growing body of evidence challenges this unidimensional view. In certain contexts, CSCs and dormant DTCs demonstrate a marked preference for OXPHOS. Glioma stem cells (85) and CD34^+^ leukemic stem cells (86), for example, exhibit heightened mitochondrial respiration mediated in part by the STAT3/MYC/SLC1A5 signaling axis (87). The TME also plays a decisive role in shaping the metabolic state of CSCs. PDAC cells exposed to hepatic stellate cells, which simulate normal liver stroma, upregulate succinate dehydrogenase subunit B (SDHB) and adopt an oxidative, quiescent phenotype. In contrast, exposure to hepatic myofibroblasts that mimic an inflammatory liver environment reactivates proliferation and drives a glycolytic shift (88). Such findings highlight that CSCs and/or dormant cells possess context-dependent metabolic plasticity, shifting between glycolytic and oxidative states in response to microenvironmental cues.

## 4 Molecular cues responsible for dormancy induction in DTCs

### 4.1 Key genetic alterations

Tumor dormancy is strongly influenced by genetic alterations. DTCs that enter a slow-cycling or growth-arrested state activate intrinsic dormancy programs (quiescence and/or senescence) that allow them to survive within the new microenvironment and escape treatments directed against proliferating cells. A key player in this process is the *FBXW7* gene encoding the F-box/WD repeat-containing protein 7 (FBXW7). FBXW7 is a component of a Skp1-Cul1-F box-type (SCF-type) E3 ubiquitin ligase, that recognizes the substrate. It regulates the cell cycle by promoting the ubiquitylation and proteasomal degradation of proteins that promote transition through the cell cycle, including cyclin E and c-Myc, thus restraining cell cycle progression. High expression of FBXW7 has been observed in various stem cells, where it promotes dormancy by inhibiting cell cycle entry. Depletion of FBXW7 induces tumor cells to exit from quiescent state in xenograft and allograft mouse models (89). Furthermore, in a study of head and neck squamous cell carcinoma (HNSCC), the paired-related homeobox transcription factor PRRX1, known for its role as a transcriptional coactivator involved in the EMT, has been implicated in regulating dormancy mechanisms. Specifically, PRRX1 has been shown to suppress miR-642b-3p levels, which are critical in mediating HNSCC cell dormancy through the TGF-β2 and p38 signaling pathways. This study demonstrated that the induction of EMT by PRRX1 may facilitate the maintenance of cancer cells in a dormant state (90). Kisspeptin-1 (KISS1), a well-known metastasis suppressor protein, has been shown to markedly reduce pulmonary and intraperitoneal metastases in xenograft models of multiple cancers, such as melanoma, breast, and ovarian cancers. The anti-metastatic effects of kisspeptin, which is the protein product of the *KISS1* gene, are proposed to occur through its binding to the G protein-coupled receptor GPR54 (91). The *NME* gene family also exhibits metastasis-suppressive functions, with *NME1* and *NME2* encoding nucleoside diphosphate kinases (NDPKs) that participate in nucleotide metabolism. Overexpression of NME1 and NME2 has been shown to significantly reduce the metastatic potential of various tumor cell lines derived from multiple histological origins, in both experimental and spontaneous mouse models of metastasis (92, 93). Studies revealed that the metastasis-suppressive effects of NME1 and NME2 are associated with its interaction with dynamin 2 (DNM2) and the subsequent promotion of DNM2 oligomerization. This interaction enhances the endocytosis of cell surface receptors and other proteins, potentially modulating their availability and downstream signaling pathways that regulate cell motility, migration, and metastatic dissemination, as evidenced through co-immunoprecipitation studies in MDA-MB-231T and MDA-MB-435 transfectant model systems (94). Multiple studies have shown that CD82/KAI1 serves as a key metastasis suppressor in various solid malignancies, such as renal (95), prostate (96), breast (97), and hepatocellular cancers (98). Similarly, MKK4 has emerged as another metastasis suppressor that limits metastatic colonization by inducing cell cycle arrest in DTCs. In ovarian cancer, ectopic MKK4 expression reduces proliferation through p38-mediated upregulation of p21 (99), whereas in prostate cancer, it exerts its effects primarily via the JNK signaling pathway (100). Despite these context-dependent signaling differences, both models align in promoting a G1 phase arrest, suggesting that cell cycle regulators such as p21, p27, p16, and cyclins D/E act as key mediators of MKK4-dependent metastasis suppression.

### 4.2 Key epigenetic factors

Transitions between proliferative and dormant states in DTCs may also result from epigenetic reprogramming, which involves transcriptional regulation via alterations in chromatin architecture. These mechanisms can include non-coding RNAs, although most of their effects are mediated through histone modifications and DNA methylation (101). A few key examples are cited below to highlight the epigenetic mechanism(s) and the dormancy-related consequences. The orphan nuclear receptor NR2F1 shows markedly reduced expression in several malignancies, including HNSCC, prostate, lung, and breast cancer, compared to normal tissues (102–104). However, NR2F1 exhibits increased expression in models of dormancy. This overexpression of NR2F1 has been associated with the dormancy of DTCs in prostate and HNSCC patients (103). Cell quiescence induced by NR2F1 is regulated by transcription factor SOX9, and retinoic acid receptor β (RARβ). Moreover, NR2F1 promotes chromatin repression by recruiting co-repressor complexes and chromatin-modifying enzymes that regulate histone acetylation and DNA methylation, while also upregulating NANOG, which is linked to the dormancy of DTCs in the bone marrow (102). This study notes that NR2F1-induced dormancy involves upregulation of certain markers shared with senescence (such as p16), but occurs without the full SASP or permanent growth arrest. In ER+ breast cancer, mitogen- and stress-activated protein kinase-1 (MSK1) plays a key role in maintaining dormancy. Low levels of MSK1 have been linked to early metastatic progression, as shown by gene expression microarray and immunohistochemistry analyses of patient tumor samples. Functionally, MSK1 maintains dormancy at secondary sites such as bone by promoting luminal cell differentiation. Mechanistically, MSK1 acts downstream of the stress-responsive kinase p38 MAPK to epigenetically regulate transcription factors like GATA3 and FOXA1 by modulating chromatin at their promoter sites. Loss of MSK1 impairs luminal differentiation, facilitates metastatic outgrowth, and enables dormancy escape (105).

### 4.3 Critical determinants of microenvironmental signaling

**Figure 3:**
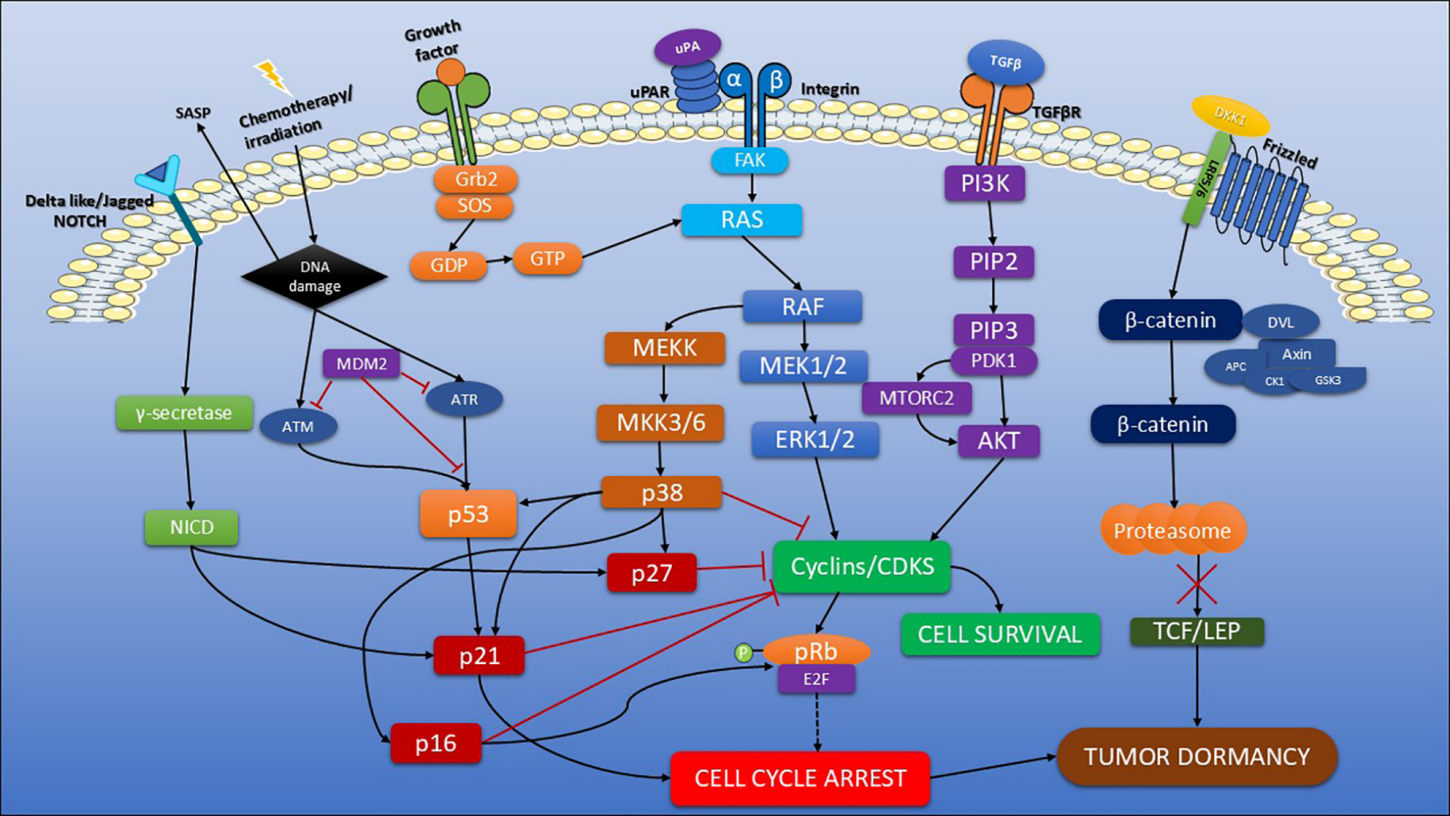
Key signaling pathways governing tumor dormancy. This schematic illustrates central molecular pathways involved in the induction and maintenance of tumor dormancy under microenvironmental cues. The ERK/p38 MAPK axis plays a pivotal role in determining whether cells enter a proliferative or dormant state. Activation of ERK1/2 via the RAS-RAF-MEK cascade promotes cell cycle progression by upregulating cyclins. In contrast, stress-induced p38 activation through MKK3/6 signaling leads to increased expression of CDK inhibitors such as p21 and p27, resulting in cell cycle arrest and promoting tumor dormancy. The PI3K/Akt/mTOR pathway, typically triggered by growth factors like TGF-β binding to their receptors, initiates a signaling cascade involving PDK1 and mTORC2 that activates Akt. Akt promotes cell growth, protein synthesis, and survival. However, under stress or hypoxic conditions, this pathway is modulated, mTOR activity decreases and autophagy is enhanced, promoting a dormant phenotype characterized by low proliferation and increased stress tolerance. Chemotherapy or irradiation can cause DNA damage, which in turn activates the p53/p21 pathway, leading to cell cycle arrest and the induction of cellular senescence, while also facilitating the release of SASP factors. Notch signaling is initiated when ligand binding induces proteolytic cleavage of the Notch receptor, releasing the Notch intracellular domain (NICD), which translocates to the nucleus to regulate target genes. Sustained Notch2 signaling may promote cellular quiescence by inducing the expression of genes involved in cell cycle arrest, contributing to the maintenance of dormancy in a context depended manner. The Wnt/β-catenin pathway plays a crucial role in regulating cell fate, proliferation, and dormancy. In the absence of Wnt ligands, β-catenin is targeted for proteasomal degradation via a destruction complex comprising Axin, APC, GSK3β, and CK1. Wnt activation inhibits this complex, allowing β-catenin stabilization and nuclear translocation, where it activates genes promoting proliferation and survival. However, low or intermittent Wnt signaling reduces β-catenin levels, leading to downregulation of key cell cycle regulators, thereby promoting cell cycle arrest and quiescence.

During cancer development, tumor cells interact with various cellular stresses and signals within the TME. Various factors have been proposed to modulate signaling pathways that regulate the balance between cell dormancy and proliferation as shown in **Figure 3** (**Table 2**). A key mechanism associated with the dormant phenotype involves the crosstalk between p38 and extracellular signal-regulated kinases (ERK1/2), components of the mitogen-activated protein kinase (MAPK) signaling pathway. The urokinase plasminogen activator receptor (uPAR) plays a crucial role in controlling the switch between tumor cell dormancy and proliferation by modulating the balance between ERK and p38 signaling pathways. High uPAR levels maintain elevated ERK activity through the activation of alpha-5 beta-1 integrin, creating a positive feedback loop that sustains cancer cell proliferation. In contrast, the downregulation of uPAR promotes a shift toward dormancy by reducing ERK activity and increasing p38 MAPK activity (113).

**Table 2:** Major microenvironmental signaling pathways in tumor dormancy.

Signaling axis/pathway	Principal effectors/components	Mechanistic role in dormancy	References
ERK-p38 MAPK axis	RAS, RAF, MEK, MKK3/6, p38, ERK 1/2	High p38/low ERK shift causes quiescence	(106)
Wnt/β-catenin	Axin, APC, GSK3β, and CK1	Low or intermittent Wnt signaling, via DKK1-mediated autocrine inhibition, keeps LCC cells dormant by downregulating β-catenin target genes that control cell cycle progression	(107)
PI3K-AKT/mTOR	PI3K, AKT, PDK1 mTOR,	Reduced phosphorylation of Akt leads to the upregulation of CDK inhibitors p21 and p27, inducing cell cycle arrest	(108)
DNA Damage Response (ATM-ATR-p53 axis)	ATM, ATR, MDM2, p53, p21	DNA damage or stress activates ATM/ATR-p53, enforcing G1 arrest and dormancy through p21	(109, 110)
Notch 2	Notch receptors, Jagged, Delta-like ligands	Sustained Notch activation induces cell cycle arrest by inducing p21^waf1/cip1^ and p27^kip1^ in a context dependent manner	(111, 112)

Similarly, the Wnt/β-catenin signaling cascade, well recognized for its involvement in regulating cell proliferation, differentiation, and survival (114), has been increasingly linked to the regulation of tumor dormancy and recurrence (115). Low or intermittent Wnt signaling, via DKK1-mediated autocrine inhibition, keeps latency-competent cancer (LCC) cells dormant by downregulating β-catenin target genes that control cell cycle progression. Malladi et al. (107) demonstrated this using *in vivo* metastasis models and gene expression analyses in LCC cells derived from early-stage breast and lung cancer cell line. Notably, reduced pathway activity leads to decreased expression of key cell cycle regulators such as c-MYC and cyclin D1 (114, 116), thereby inhibiting cell cycle progression and promoting quiescence.

The PI3K/Akt/mTOR signaling pathway intricately governs tumor cell dormancy. This pathway is vital for regulating cell growth, metabolism, and survival. When this pathway is dysregulated, it can contribute to cancer development, progression, and recurrence (117). Akt, in particular, plays a key role in promoting cell survival by blocking pro-apoptotic signals. This anti-apoptotic function helps dormant cancer cells avoid programmed cell death and remain viable over time. Notably, reduced phosphorylation of Akt leads to the upregulation of CDK inhibitors p21 and p27 (108, 118). This, in turn, inhibits the formation of CDK-cyclin complexes, blocking the transition from the G_0_/G_1_ to S phase, thereby inducing cell cycle arrest and promoting entry into a dormant state (119). In parallel, autophagy functions as an essential survival mechanism that removes damaged organelles, misfolded proteins, and other cytosolic debris, thereby recycling cellular components and maintaining energy balance under metabolic stress (120). Dormant DTCs exhibit higher levels of autophagy compared to proliferating cells, as they activate autophagic pathways to survive oxidative stress and improve cellular bioenergetics (121). Inhibition of ATG5 and ATG7 expression in dormant breast cancer cells leads to reduced survival, indicating that autophagy supports dormancy maintenance (122). Furthermore, patient-derived ovarian tumor samples have shown a four-fold rise in autophagy markers when comparing primary tumors to dormant lesions observed after chemotherapy recurrence (123). The re-expression of the tumor suppressor gene ARHI (DIRAS3), which is frequently downregulated in ovarian cancer, promotes autophagy by inhibiting the PI3K/AKT/mTOR pathway and upregulating ATG4. This re-expression leads to autophagic cell death in culture but enables dormancy in xenograft models (124). Additionally, alongside the inhibition of the PI3K/AKT/mTOR pathway, dormant cancer cells exhibit activation of AMPK, thereby enhancing autophagy to support survival under stress conditions (125). The mTOR complexes, particularly mTORC1, serve as crucial regulator of both autophagy and metabolic reprogramming. Under nutrient-rich conditions, mTORC1 inhibits autophagy (126), while AMPK has traditionally been considered a promoter of autophagy in response to energy deprivation. However, recent *in vitro* human cell and *in vivo* mouse model system studies challenge this conventional view, revealing a more intricate regulatory mechanism for AMPK. New evidence demonstrates that AMPK can suppress autophagy by inhibiting ULK1 activity through phosphorylation at Ser556 and Thr660. This phosphorylation event strengthens the AMPK-ULK1 association, thereby preventing ULK1 activation under amino acid deprivation and simultaneously protecting ULK1 and associated autophagy regulators from caspase-mediated proteolysis. By inhibiting abrupt autophagy induction while preserving essential autophagy components, AMPK ensures that autophagy can be swiftly reinitiated once the energy stress subsides, thus maintaining cellular homeostasis (127).

### 4.4 Role of immune system

The immune system, mainly the immune cells in the TME also plays a critical role in the induction and maintenance of tumor dormancy. Effector T cells, particularly CD8+ and CD4+ subsets, play key roles in cancer dormancy. CD8+ T cells are potent tumor suppressors due to their ability to recognize intracellular tumor antigens. CD4+ T cells contribute by directly killing tumor cells or modulating the tumor microenvironment (128). In a mouse model, depletion of CD8+ T cells led to a shorter duration of dormancy and earlier recurrence of B cell lymphoma in the spleen, indicating that CD8+ T cells help induce and maintain dormancy, primarily through interferon-γ (IFN-γ) production (129). Likewise, in a melanoma mouse model, cytostatic CD8+ T cells played a role in maintaining dormancy, as removing these cells led to a more rapid proliferation of tumor cells (130). In a breast cancer mouse model, CD39^+^PD-1^+^CD8^+^ T cells were reported to induce metastatic dormancy in the lungs. These cells, exhibiting features of both effector function and exhaustion, secrete TNF-α and IFN-γ, which possibly plays a role in the induction of senescence in cancer cells (131). Furthermore, the removal of natural killer (NK) cells appears to create a conducive environment for metastatic progression. NK cells sustain dormancy of DTCs by secreting IFN-γ. In the absence of NK cells, dormant Lewis lung carcinoma cells resume growth, leading to metastatic outgrowth in secondary organs such as the liver in an *in vivo* mouse model (132). In a HT29 human colon cancer xenograft mouse model, NK cells were shown to play a crucial role in controlling both primary tumor growth and distant metastasis. Computational modeling further suggested that, perforin-mediated cytotoxicity by NK cells can cytolyze HT29 human colon cancer cells and suppress the proliferation of DTCs, thereby maintaining them in a dormant state for at least 30 days (133). Moreover, the most common immune evasion mechanisms include lack of expression of Major Histocompatibility Complex I (MHC I) and tumor antigens. For instance, analyses of liver tissues from both murine models and patients with pancreatic ductal carcinoma revealed the presence of DTCs lacking MHC I and cytokeratin 19 (CK19) expression. This indicates an alternative immune escape strategy, whereby these cancer cells evade detection and killing by T-cells, potentially through endoplasmic reticulum (ER) stress-related mechanisms (134).

### 4.5 Role of hypoxia and cancer therapy

Hypoxia is recognized as a critical determinant of tumor dormancy. A hypoxic TME, characterized by oxygen depletion and disrupted homeostasis, is a hallmark of most solid tumors (135). Under hypoxic conditions, cancer cells enter and maintain a dormant state through the action of transcription factors like hypoxia-inducible factor 1 α (HIF-1α). Commonly, HIF-1α promotes a metabolic shift toward glycolysis (136), allowing cells to adapt and survive under low-oxygen conditions. This shift not only sustains cell viability during dormancy but also presents an opportunity to the cells for swift proliferation when favorable conditions return. In a salivary adenoid cystic carcinoma (SACC) mouse model, CoCl_2_ induced hypoxia promotes reversible tumor dormancy through upregulation of the transcription factor DEC2. Under low-oxygen conditions, DEC2, often co-expressed with HIF-1α, triggers G_0_/G_1_ cell cycle arrest and activates a Slug-mediated EMT program, leading to suppressed proliferation and increased invasiveness. When these dormant, DEC2-high cells reach oxygen-rich environments such as the lungs, DEC2 expression decreases, allowing the cells to exit dormancy and form metastatic outgrowths (137). More recently, Dai et al. (138) further elucidated this mechanism by identifying a regulatory miR-922/DEC2 axis, in which miR-922 directly targets DEC2 to negatively regulate its expression.

Another contributor to angiogenic dormancy is the dysregulation in the balance between pro-angiogenic and anti-angiogenic factors, which tilts towards factors that enhance anti-angiogenic elements, thereby inhibiting new blood vessel formation. Key molecules involved in this anti-angiogenic state include TSP-1, endostatin, and angiostatin (139). These factors exert their effects by suppressing endothelial cell proliferation and migration, processes essential for neovascularization. An experimental study employing a xenograft model in SCID mice implanted with U-87 human glioblastoma cells provided evidence supporting the mechanism of TSP-1 (140). Research findings revealed that quiescent U-87 cell populations developed smaller, poorly vascularized neoplastic lesions, whereas proliferative variants formed larger, highly vascularized tumors. Significantly, the quiescent populations demonstrated increased TSP-1 expression, a protein recognized for suppressing vessel formation and restricting tumor invasion. These observations indicate that enhanced TSP-1 activity in quiescent populations contributes to angiogenesis suppression, resulting in delayed tumor development. This mechanism explains how increased TSP-1 concentrations can modify the equilibrium toward vessel-inhibiting factors, maintaining neoplasms in a quiescent, non-vascularized condition (116).

Notch signaling pathway also plays key role in regulating tumor dormancy. In endothelial cells, Notch ligand DII4 has been linked to the escape of colorectal and T-cell acute lymphoblastic leukemia (T-ALL) cells from dormancy, aligning with its role in promoting tumor angiogenesis and activating the Notch signaling pathway. The presence of DII4 is increased in aggressive tumors but absent in quiescent ones, as shown in NOD/SCID mouse xenograft models using T-ALL cell lines and *ex vivo* analyses such as RT-PCR, Western blotting, and immunofluorescence, suggesting its role in tumor dormancy (141). Angiogenic signals within endothelial cells can activate Notch3, which may promote tumor proliferation. Notch3 has been implicated in the regulation of MAP kinase phosphatase-1 (MKP-1). Interestingly, while Notch3 and MKP-1 expression is typically downregulated in dormant tumors (142), these dormant cells often exhibit elevated levels of phosphorylated p38 MAPK. This suggests that tumor dormancy can be influenced by angiogenesis-related mechanisms, particularly through the involvement of Notch signaling pathways (135).

Cancer treatment itself can induce drug resistance and dormancy in tumor cells, eventually causing therapy failure. Cells that acquire resistance in response to therapy often exhibit a transient, slow-proliferating or dormant state, while still maintaining viability. This phase typically relies on the continued presence of cytotoxic agents and can be reversed when the drug is removed. It has been proposed that the dosage and duration of cancer treatment play a critical role in determining the fate of tumor cells. Depending on these factors, tumor cells may enter a state of quiescence, undergo senescence, or activate pro-apoptotic pathways leading to cell death (143). Imatinib treatment in gastrointestinal stromal tumors induces quiescence (144), and shifts their metabolism from glycolysis to oxidative phosphorylation (145). Tamoxifen-resistant (TAMR) breast cancer cell lines were established to model the delayed recurrence associated with prolonged hormone therapy (146). The motility of these cells, a key factor in tumor metastasis, was assessed through live-cell imaging and manual tracking using ImageJ. TAMR EFM19 cells exhibited a marked reduction in migration speed compared to their parental EFM19 counterparts. Moreover, parental EFM19 cells displayed significant intercellular gap closure over 48 hours, while TAMR EFM19 cells showed reduced wound healing capacity. These observations suggest that extended hormone treatment may impair cellular motility, potentially contributing to the induction of cancer dormancy (146).

## 5 Molecular cues responsible for reactivation of dormant DTCs

Metastatic cancer cells often spread from the primary tumor to distant organs, where they may remain dormant and clinically undetectable for long periods. Upon reaching these secondary sites, DTCs often encounter a new and unfavorable microenvironment, which can either drive them into quiescence or induce a senescent state. During dormancy, DTCs can accumulate genetic and epigenetic changes, enabling them to adapt more effectively to their new microenvironment. Eventually, a subset of these cells may escape dormancy and “reawaken”, leading to the formation of expanding metastatic masses (147). The precise signals behind this reactivation are currently being explored, as they are believed to play a crucial role in the transition from dormancy to active tumor growth.

### 5.1 Role of tumor microenvironment

Alterations in the TME have been proposed to potentially trigger the escape of DTCs from their dormant state. The coordinated interplay among diverse stromal cell type including macrophages, myeloid-derived suppressor cells, neutrophils, pericytes, fibroblasts, and vascular endothelial cells, plays a critical role in tumor recurrence. This complex interaction is mediated through a network of cytokines, growth factors, and chemokines (148). For example, in triple-negative breast cancer, tissue-resident macrophages within the mammary gland act as an important source of tumor-associated macrophages (TAMs), playing a significant role in driving both local tumor relapse and distant metastatic spread (149). Furthermore, In hepatocellular carcinoma, CXCL10/TLR4-mediated MMP14 activation promotes recruitment of monocytic myeloid-derived suppressor cells (MDSCs) after acute liver injury, fueling post-transplant HCC relapse, as evidenced by clinical data, animal studies, and cell-culture assays (150). Moreover, integrin receptors, particularly β1 integrins, play a crucial role in both initiating and sustaining tumors by suppressing p53-driven dormancy checkpoints. By mediating adhesion to the ECM, β1 integrin signaling regulates cell cycle entry, survival, and migration. Its activation, together with intracellular modifications and alterations in the TME, drives dormant cells to resume proliferation and initiate metastatic relapse (151, 152). Disruption of this signaling pathway, either through downregulation of the uPA-uPAR interaction or antibody-mediated inhibition of β1 integrin, has been shown to induce cancer cell dormancy, as demonstrated in an *in vivo* chick CAM model (106). However, removing this inhibition can reverse the dormancy and restart cancer cell proliferation. Reduction of myosin light chain kinase (MLCK) expression in MCF10A breast epithelial cells has been shown to induce cellular senescence, characterized by diminished proliferation and enhanced migration. This reduction activates a p53 (TP53) and AKT-mTOR-dependent upregulation of p21, driving the senescence process. Additionally, p21 elevation stimulates the release of senescence-associated cytokines, which further enhance cell migration in MLCK-deficient MCF10A cells (153). These findings suggest that MLCK may regulate the balance between cell proliferation and migration during breast cancer progression.

TSP-1, secreted by endothelial cells in a stable microvascular environment, plays a critical role in sustaining cancer cell quiescence (154), and senescence (155). However, during the onset of neovascularization, the inhibitory influence of TSP-1 is reduced, and its function shifts toward supporting cancer cell proliferation, thereby markedly enhancing tumor progression (154). Similarly, osteopontin has been recognized as a critical contributor to breast cancer recurrence by facilitating the recruitment of macrophages into the TME. In response to tumor-derived interleukin-4 (IL-4), osteopontin promotes the polarization of macrophages toward a pro-tumorigenic phenotype, characterized by elevated expression of IL-4 receptors and arginase-1. These osteopontin-activated macrophages have been shown to support tumor recurrence by enhancing cancer cell proliferation (156). In addition, hepatic stellate cells have been reported to release soluble mediators that promote the growth of breast cancer cell lines such as MCF7 and MDA-MB231 in a co-culture experiment. Elevated levels of IL-8 and MCP-1 were observed in secretions from both activated stellate cells and primary human non-parenchymal liver cells. IL-8 was shown to alleviate growth arrest caused by serum deprivation in MDA-MB231 cells and to enhance tumor cell proliferation in *ex vivo* human 3D model system (157).

Diet and medications may play a significant role in regulating tumor growth and dormancy. Poor dietary habits that result in increased adipose tissue and obesity have been linked to breast cancer recurrence. Adipocytes contribute to inflammation by releasing MMP11 and pro-inflammatory cytokines such as IL-6 and IL-1β. Additionally, adipose tissue is associated with estrogen production, which can promote the proliferation of tumor cells (158).

### 5.2 Role of inflammation and wound healing in awakening of dormant cells

Chronic inflammation has been strongly linked to cancer recurrence, particularly in cases of endometrial (159), oral (160), colorectal (161), HCC (162) and breast cancers (163, 164). All stages of the invasion-metastasis cascade trigger inflammation and involve the breakdown and remodeling of the ECM, a process central to wound healing. Wound healing itself follows three phases: inflammation, regeneration, and remodeling. During the inflammation phase, damage to epithelial cells releases inflammatory signals, which recruit immune cells, such as neutrophils, to the wound site. These immune cells prevent infection, clears damaged tissue and initiates the repair process. Inflammation can be classified into two types: Type I and Type II. Type I inflammation involves the production of pro-inflammatory cytokines, which are crucial for initiating immune responses against infections or cancer cells. Conversely, Type II inflammation produces anti-inflammatory cytokines that promote cell proliferation and aid in wound healing. Typically, after Type I inflammation has fulfilled its role, it transitions into Type II. However, when Type I inflammation becomes chronic, it can promote cancer by activating the STAT3 pathway, inducing oxidative DNA damage, and leading to mutations. While Type I inflammation can help maintain tumor dormancy by suppressing cell proliferation, chronic inflammation combined with Type II elements may trigger wound healing mechanisms, potentially reactivating dormant tumor cells and leading to cancer relapse (165).

An epidemiological study identified a subgroup of breast cancer patients who experienced early recurrences, marked by multiple, similarly sized metastases, suggesting a synchronized onset of growth. It is hypothesized that post-surgical wound healing may act as a synchronizing signal for tumor relapse. Studies into delayed breast reconstruction revealed a peak in relapses 18 months after surgery (166), a pattern similar to that seen following primary breast cancer surgery (167). The severity of these relapses was directly correlated with the extent of surgery, establishing a dose-response relationship that suggests a causal link between surgical wound healing and cancer recurrence (166).

## 6 Therapeutic approaches against tumor dormancy

**Figure 4:**
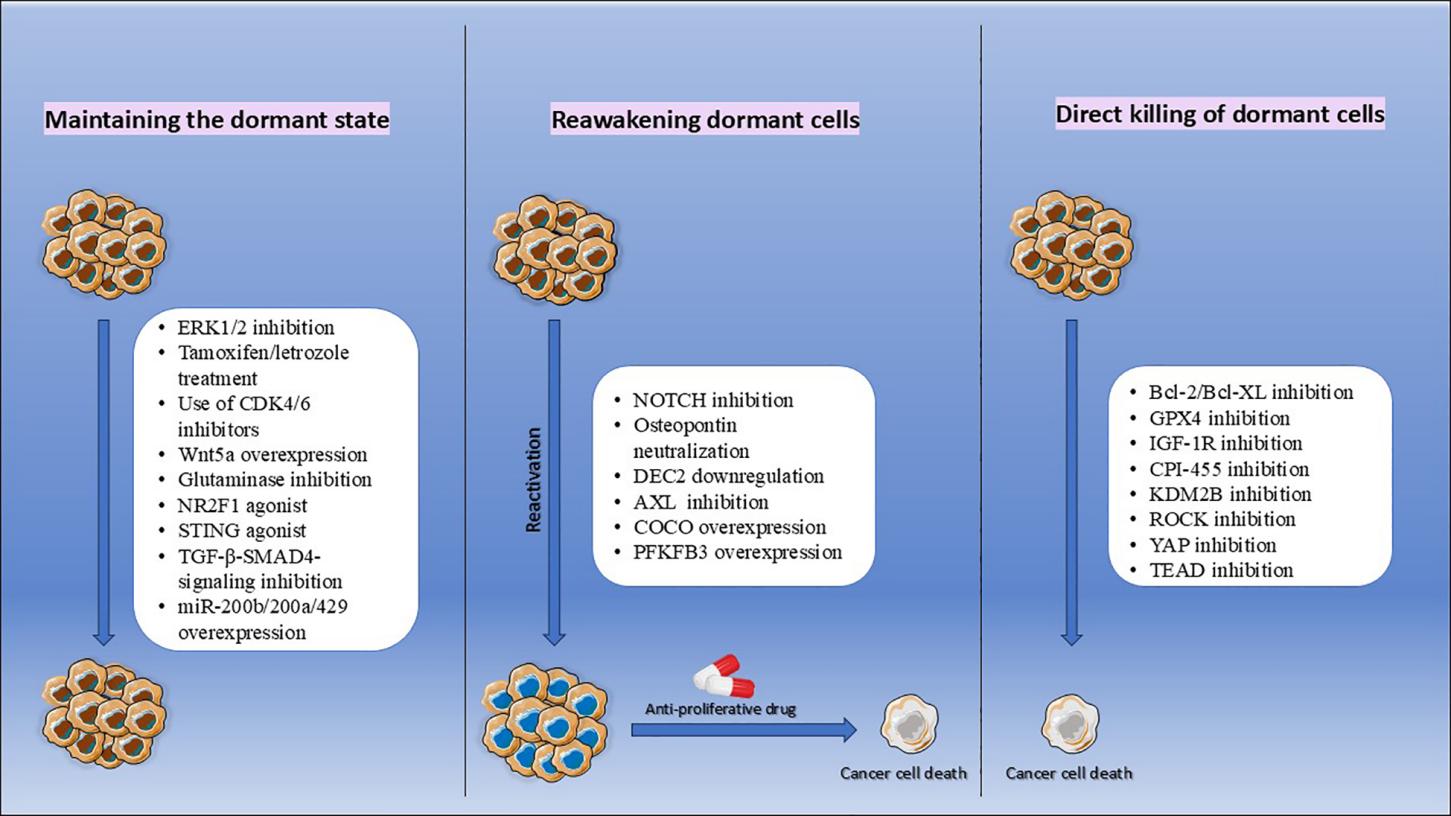
Therapeutic strategies for targeting dormant cancer cells. The illustration presents three proposed approaches to eradicate dormant disseminated tumor cells (dDTCs) and prevent metastatic relapse. (1) Maintenance strategy: Sustaining dormancy by inhibiting pathways that support proliferation and metastatic outgrowth, thereby preventing tumor regrowth. (2) Reactivation strategy: Intentionally reawakening dormant cells to a proliferative state, rendering them susceptible to antiproliferative therapies aimed at eliminating actively dividing cells. (3) Direct elimination strategy: Targeting quiescent dormant cells directly through mechanisms such as targeting autophagy pathways and/or apoptosis induction to prevent future reactivation and recurrence.

Dormant tumor cells coexist with fast proliferating cells even during cancer progression and may contribute to therapeutic resistance. The timing, mechanism, and approach to treating these dormant cells remain largely unclear. Conventional therapies primarily target rapidly dividing cells, leaving dormant cells resistant to treatment (146). Therefore, understanding the mechanisms controlling the switch between dormancy and proliferation is essential for developing new therapeutic approaches. Suggested therapeutic strategies for targeting dormant tumor cells are illustrated in **Figure 4**.

### 6.1 Maintaining the cells in dormant state

A key therapeutic strategy involves maintaining tumor cells in a permanent state of dormancy by keeping residual cells inactive and non-proliferative. Dormant cells are typically marked by elevated p38 MAPK activity and reduced ERK1/2 activity, which serve as the major markers of dormancy (106). Modulating the p38/ERK balance (by the site-specific targeting of senescent and/or quiescent cells possibly by using Stimuli-responsive smart drug release systems (SDDS)) could potentially induce permanent growth arrest in tumor cells, thereby preventing tumor recurrence and metastasis. The use of CDK4/6 inhibitors such as palbociclib (168), abemaciclib (169), and ribociclib (170), which are crucial in regulating the transition of the cell cycle, could help to keep cancer cells in senescent or quiescent state. Additionally, agents like TSP1 have demonstrated potential in reducing the proliferation of invasive ductal carcinoma cells (171).

Various preclinical targets have shown potential in inhibiting DTC growth, as summarized in **Table 3**, alongside approved therapeutic agents. For instance, Wnt5a directly inhibits proliferative Wnt/β-catenin signaling pathways, inducing dormancy in prostate cancer cells in both *in vitro* co-culture and *in vivo* mouse model. This effect is mediated by the induction of Siah E3 Ubiquitin Protein Ligase 2 (SIAH2), which suppresses Wnt/β-catenin signaling, and is dependent on receptor tyrosine kinase-like orphan receptor 2 (ROR2) (172). In another *in vitro* study, an increase in non-dividing quiescent cells was observed when C4-2B4 prostate cancer cells were incubated with bone-secreted factors such as recombinant human DKK3 and BMP1 (173). Furthermore, another therapeutic approach currently being explored involves the use of a DNA methylation inhibitor - 5-azacytidine (AZA), in combination with retinoic acid receptor ligands, such as all-trans retinoic acid (atRA) or AM80, an RARα-specific agonist to promote stable dormancy in cancer cells. This treatment strategy, applied to HNSCC or breast cancer cells, activates a SMAD2/3/4-dependent transcriptional pathway that reestablishes TGFβ signaling, leading to an anti-proliferative effect. Notably, both AZA combined with atRA and AZA combined with AM80 have shown significant potential in inhibiting the formation of lung metastases in HNSCC by inducing and maintaining solitary DTCs in a SMAD4^+^/NR2F1^+^ dormant state, as demonstrated by *in vitro* cell line and *in vivo* mouse models (174).

**Table 3:** Gene/pathways responsible for the sleeping strategy.

Target gene/signaling pathway	Mechanism of target	Cancer model	Experimental methodology	Sample source	References
CDK4/6	Inhibition (palbociclib)	Liver cancer	Ki-67 immunostaining (cell proliferation); SA-βGal staining (senescence); Flow cytometry with DAPI (G_0_/G_1_ arrest); Western blot for p-Rb and cell cycle regulators; Bioluminescence imaging (*in vivo* tumor growth).	HCC cell lines (BT549, GP2-293, HCC202, HepG2, Huh1, Huh7, Hep3B, JHH2, Alexander, JHH7, Li7, PLC/PRF/5, skHep1, SNU423, SNU387, SNU398, SNU449, SNU475), *ex vivo* organotypic culture of patient-derived HCC tissues, xenograft mouse model (NCR-NU-NU, C57BL6), Human patient samples	(168)
	Inhibition (Abemaciclib)	Breast cancer	TR-FRET kinase assay for CDK4/6-CyclinD activity (Rb phosphorylation); Filter binding assay; IC_50_ and dose-response analysis for inhibition; shRNA-mediated CDK4/6 knockdown with Western blot validation; *In vitro* washout assay to assess Rb phosphorylation durability.	Human breast cancer cell lines (HCC1419, BT-474, UACC-3133, ZR-75-30, MDA-MB-361, CAMA-1, EFM-19, MDA-MB-415, MCF-7, T-47D, MDA-MB-175-VII ZR-75-1, UACC-812, MDA-MB-134-VI, AU-565, HCC1569, HCC202, HCC1954, HCC2218, MDA-MB-453, SK-BR-3, UACC-3199, UACC-893, UACC-2087, DU-4475, BT-20, BT-549, HCC38, HCC70, HCC1143, HCC1187, HCC1395, HCC1806, HCC1937, Hs-578-T, MDA-MB-157, MDA-MB-231)	(169)
	Inhibition (Ribociclib)	Breast cancer	Phase III clinical evaluation of ribociclib + endocrine therapy (fulvestrant/letrozole) in HR^+^/HER2^-^ ABC patients; tumor response assessed by CT, mammogram, ultrasound, and bone scan per RECIST v1.1; safety monitored via CTCAE v4.0; histopathological assessment post-therapy; patient-reported outcomes and QoL evaluations included.	Human patient sample (analysis of clinical information)	(170)
Wnt5a	Overexpression	Prostate cancer	Ki-67 immunostaining for proliferation; Flow cytometry for G0/G1 arrest; DiD dye-retention assay for dormancy; Western blot for p21, p27, β-catenin, SIAH2; Genetic manipulation via *Wnt5a* knockdown and ROR2/SIAH2 silencing; *In vivo* validation using DiD-labeled, luciferase-expressing PCa cells injected into mouse tibiae; Bioluminescence imaging and bone lesion scoring for metastasis evaluation.	Human prostate cancer cell line (PC-3, C4-2B, and patient-derived primary PCa cells), xenograft mouse model (BALB/c-nu nude)	(172)
ERK	Mek1/2 inhibition (PD98059)	Head and neck carcinoma, fibrosarcoma, melanoma, prostate cancer, breast cancer	PD98059 (25–50 µM) treatment suppressed ERK phosphorylation confirmed by Western blot and GAL4–Elk1 luciferase reporter assay; dormancy validated by growth arrest in CAM assay; pull-down assays for Rac/Cdc42 activity and immunoprecipitation of integrin–uPAR complexes assessed signaling changes; immunofluorescence visualized cytoskeletal reorganization.	Human cancer cell lines (HEp3, HT1080, M24met, PC3, MDA-MB-453, MCF-7, MDA-MB-231, MDA-MB-468)	(106)
uPAR/α5β1 signaling complex	anti-uPAR antibody R2 based inhibition	Head and neck carcinoma, fibrosarcoma, melanoma, prostate cancer, breast cancer	Treatment with anti-uPAR antibody (R2) disrupted the uPAR/α5β1 integrin complex, assessed using fibronectin fibril formation assay, immunofluorescence microscopy, Western blot for phospho-ERK inhibition, and co-immunoprecipitation of surface-biotinylated proteins. *In vivo* confirmation by CAM tumor growth assay.	Human cancer cell lines (HEp3, HT1080, M24met, PC3, MDA-MB-468, MCF-7, MDA-MB-231, MDA-MB-453)	(106)
DKK3, BMP1	Overexpression	Prostate cancer	DKK3 and BMP1 treatment in cancer cells; dormancy confirmed by live-cell imaging and co-staining for Ki-67^-^/p27^+^; p-p38 nuclear translocation assessed by IF.	Human prostate cancer cell line (PCa C4-2B4 and C4-2b)	(173)
NR2F1	Agonist (C26)	HNSCC	Cells treated with C26; dormancy confirmed by qPCR, IF, RNA-seq of FACS-sorted GFP^+^ cells showing quiescence and neural crest gene upregulation; NR2F1 knockout nullified effects; CAM assay showed growth arrest without apoptosis.	Human cell lines (T-HEp3, FaDu, SQ20B), Patient-derived xenograft (PDX) mouse models, patient-derived organoids in 3D culture, and *in vivo* CAM model	(103)
TGF-β-SMAD4- signaling	Inhibition (AZA+atRA or AZA+AM80)	HNSCC	Treatment with AZA+atRA or AZA+AM80; RNA-seq and ChIP-seq to assess transcriptional and enhancer changes; immunofluorescence and Western blot for NR2F1, p27, SMAD2/3/4; SMAD4 shRNA knockdown for functional validation; CAM and mouse assays to evaluate tumor growth, dormancy, and metastasis suppression.	Human HNSCC cell line (T-HEp3), *in vivo* CAM model and nude mice model	(174)
miR-200b/200a/429	Overexpression	Breast cancer	Generation of doxycycline-inducible transgenic mice co-expressing Neu/Erbb2 and the miR-200b/200a/429 cluster (MTB-TANba429). Tumor initiation and growth were monitored through palpation and imaging. Histological and whole-mount analyses were performed to evaluate ductal morphology, epithelial hyperplasia, and tumor development. Differential gene expression was assessed using RNA-seq of mammary glands at early (55-day) and late stages, with validation through TPM normalization to epithelial markers (Krt18, Krt19). Comparative histopathology confirmed suppression of Erbb2-induced transformation and maintenance of normal epithelial architecture, indicating dormancy induction by miR-200 overexpression.	*Neu/Erbb2* transgenic mice model	(175)
STING	Agonist (MSA-2, ADU-S100)	Lung cancer	STING agonists (MSA-2, ADU-S100) were used *in vitro* (qRT-PCR for CXCL10, CCL5 induction) and *in vivo* (intracardiac inoculation of cancer cells in mice, followed by agonist treatment). Disseminated tumor burden, immune cell depletion (NK, CD4^+^, CD8^+^), and survival analyses assessed STING-dependent dormancy maintenance.	Human and mouse cell lines (H2087-LCC, H2030-BrM, A549, KPad1, KP-482T1) mice model (Athymic nude mice, NSG, C57BL/6J, B6(Cg)-*Tyr^c−2J^*/J) and human patient samples	(176)
Glutaminase (NRF2-driven metabolic state)	Inhibitor (CB-839, BPTES)	Breast cancer	Glutaminase inhibition was performed using CB-839 and BPTES in primary and recurrent breast tumor cells. Cell viability and colony formation assays were used to evaluate growth inhibition and dormancy reactivation.	Human breast cancer cell lines (BT-474, SKBR3) *In vitro* mammosphere cultures (derived from primary Her2-driven tumors of doxycycline-inducible MTB/TAN mice), and Doxycycline-inducible HER2-driven mouse model (MTB/TAN)	(177)
GPC3	Overexpression	Breast cancer	Dormancy-associated signaling was assessed by Western blotting to quantify phospho-p38, phospho-Erk, p21, p27, and SOX2 expression levels. The Erk/p38 activity ratio was calculated to determine dormancy signaling balance. qPCR and Western blot were used to confirm epithelial (E-cadherin) and mesenchymal (vimentin) marker expression in reactivated “ex-dormant” cells. Phalloidin-Alexa 546 cytoskeletal staining evaluated actin organization associated with dormant morphology. *In vivo* dormancy and metastatic suppression were validated through intravenous inoculation of tumor cells in BALB/c mice, followed by lung metastasis quantification and histological analysis after p38 inhibition (SB203580 treatment).	Genetically modified murine breast cancer cell line (LM3), mouse model (LM3-GPC3 and LM3-vector cell sublines inoculated into BALB/c)	(178)
PRRX1	Overexpression	HNSCC	Subcutaneous xenograft model using PRRX1-overexpressing and vector control Cal-27 cells in nude mice to assess tumor growth suppression and dormancy; tumor volume measurement over 6 weeks; H&E and immunohistochemistry for PRRX1, TGF-β2, p38, and E-cadherin expression; Western blot and RT-PCR analyses to evaluate EMT- and dormancy-related markers (E-cadherin, TGF-β2, p38, and miR-642b-3p).	Human HNSCC cell lines (SCC-9, SCC-15, Cal-27 SCC-25), xenograft mouse model (BALB/c nu/nu), human patient samples	(90)
GILZ	Knockdown	Melanoma	siRNA-mediated knockdown of GILZ in melanoma cells induced cellular dormancy by driving cells into G_0_-phase arrest. This was accompanied by decreased sphere and colony formation, delayed tumor initiation in mice, and activation of the FOXO3A/p21CIP1 signaling axis, confirmed through qRT-PCR, western blotting, flow cytometry, and immunofluorescence.	Cell lines (A375 human and B16F1 murine), syngeneic mice model (C57BL/6), human patient samples	(179)
sFRP1	Inhibition using neutralizing antibody	Melanoma	To assess sFRP1 inhibition–induced dormancy, aged C57BL/6 mice were intradermally injected with melanoma cells. After three weeks, mice received intraperitoneal anti-sFRP1 antibody or IgG control for two weeks. Lung tissues were analyzed by H&E and IHC for MITF and Ki-67. Reduced Ki-67 and increased single MITF^+^ cells indicated dormancy induction.	Human cell lines (FS4, FS5, FS13, FS14), *in vivo* mouse model (YUMM1.7 cells injected into C57BL/6)	(180, 181)
β-adrenergic receptor signaling	β-adrenergic receptor antagonist (propranolol)	Prostate cancer	Dormancy regulation via β-adrenergic signaling was studied using a PC3–MC3T3 co-culture model. Norepinephrine (NE) induced reactivation, while propranolol (β-blocker) inhibited this effect. Dot blot and qRT-PCR assessed GAS6 expression. CREB activity and cAMP levels were measured using luciferase and biochemical assays, and ChIP confirmed reduced CREB1/ATF4 binding to the GAS6 promoter. *In vivo*, NE and propranolol-treated mice were analyzed for osteoblast GAS6 mRNA to confirm dormancy maintenance.	Human cell line (PC3), murine cell line (MC3T3-E1 subclone 4), mouse model (C57BL6/J mice)	(182)

The miR-200 family, comprising miR-200a, miR-200b, miR-200c, and miR-429, has emerged as a significant factor in breast cancer regulation. Known for maintaining the epithelial characteristics of mammary cells, reduced levels of miR-200 are commonly linked to the EMT seen in tumor progression. Thus, re-expression of the miR-200b/200a/429 cluster in claudin-low murine breast cancer cells (RJ423) led to reduced expression of EMT markers, including *Vim, Snai1, Twist1, Twist2*, and *Zeb1*. This resulted in a transition toward an epithelial-like morphology and suppressed cell proliferation *in vitro*. Additionally, this miR-200b/200a/429 cluster effectively reduced lung metastasis in an experimental model and induced a dormancy-like state in primary mammary tumors, where growth was either halted or slowed significantly. These dormant tumors also exhibited increased collagen levels and were highly vascularized, underscoring the impact of miR-200 re-expression in inducing tumor dormancy and inhibiting metastasis (183). Moreover, the miR-200 family appears to regulate stromal cells, particularly by maintaining functional myoepithelial cells, which may further prevent mammary epithelial transformation and tumor development, as shown in studies where overexpression of the miR-200b/200a/429 cluster prevented *Neu* oncogene-induced mammary tumor formation. This highlights the miR-200 family’s multifaceted role in inhibiting tumor progression (175).

Hormone-dependent cancers, particularly ER+ breast cancer, often enter a dormant state as a result of hormone-deprivation therapies. In early studies using *in vitro* breast cancer models, it was shown that depriving tumors of hormones could effectively halt their growth. Long-term oral adjuvant treatment using the ER antagonist tamoxifen or the aromatase inhibitor letrozole has become the standard approach to care. These therapies are typically administered for several years after diagnosis and have proven to significantly reduce the risk of cancer reactivation (184).

Since this therapeutic approach keeps cancer cells dormant rather than eliminating them, prolonged treatment is necessary, leading to increased toxicity and the potential for cancer cells to develop resistance.

### 6.2 Awakening of the dormant cells

The strategy of awakening dormant cells enhances their sensitivity to anticancer treatments by driving them back into a proliferative state. An approach to stimulate dormant cells into re-entering the cell cycle is by targeting the dormancy-promoting factors released by the TME. By disrupting these signals, latent cancer cells can be prompted to become active and potentially making them more susceptible to therapeutic interventions (185), as summarized in **Table 4**. For example, siRNA-mediated disruption of the APC^CDH1^-SKP2-p27^Kip1^ signaling pathway has been shown to promote exit from quiescence and enhance the efficacy of imatinib in a xenograft mouse model of gastrointestinal stromal tumors (GIST) (144). In a xenograft mouse model of human HNSCC cells, stimulation by TGF-β triggers a dormancy phenotype that is dependent on the MAPK p38α/β pathway. However, when the activity of TGF-β receptor I or p38α/β is systemically inhibited, dormant cancer cells are reactivated (30), leading to an increased metastatic spread to organs such as the liver, spleen, and bone marrow. A study demonstrated that antibody-based neutralization of osteopontin (a factor produced in the bone marrow that promotes quiescence in leukemia cells), facilitated the re-entry of dormant leukemia cells into the cell cycle. When combined with antimetabolite treatment using cytarabine, this neutralization significantly reduced the residual disease burden (186).

**Table 4:** Gene/pathways responsible for the re-awakening strategy.

Target gene/signaling pathway	Mechanism of target	Cancer model	Experimental methodology	Sample source	References
Osteopontin	Antibody-based neutralization	Lymphoblastic leukemia	Anti-OPN neutralizing antibodies were administered *in vivo*; effects were analyzed using adhesion assays, intravital microscopy, flow cytometry for GFP^+^ and F4/80^+^ cells, Ki-67 staining for proliferation, and MTT assay for *in vitro* validation.	Human cell line (Nalm-6-GFP or primary ALL cells), xenograft SCID mouse model	(186)
AXL	Inhibition (cabozantinib, BMS-777607)	Myeloma	Dormant (eGFP^+^DiD^hi^) and proliferative (eGFP^+^DiD^neg^) cell populations were quantified by flow cytometry in bone marrow and spleen. Micro-CT imaging and histomorphometric analysis evaluated osteoclast activity and bone structure, while gene expression profiling confirmed target selectivity of the inhibitors.	Cell lines (RM1 prostate cells, MC3T3 osteoblastic cells, 5TGM1-eGFP myeloma cells, and 5TGM1-luc MM cells.), syngeneic mouse model (5TGM1-eGFP myeloma cells were labeled with the lipid dye DiD and injected IV into 6- to 8-week-old C57BL/KaLwRijHsd mice)	(187)
DEC2	CXCL1 based downregulation	Oral squamous cell carcinoma	Human cytokine array identified CXCL1 upregulation in CAF-CM; validated by qRT-PCR and Western blot. CCK-8 assay assessed CXCL1-induced proliferation, and CXCR2 inhibitor (SB225002) confirmed pathway specificity. *In vivo*, DEC2^hi^ OSCC cells co-injected with CAFs into nude mice showed dormancy reactivation, verified by tumor volume measurement, IHC (DEC2, α-SMA), and qRT-PCR of dormancy markers (p21, p27, NR2F1).	Human cell line (CAL27, SCC47) and xenograft mouse model (BALB/c nude)	(188)
Notch2	γ-secretase inhibitor (dibenzazepine)	Breast cancer	Intratibial injection of luciferase-labeled MDA-MB-231 (MDALUC) cells into immunocompromised CD1 nu/nu mice to establish bone dormancy. After 4 weeks, mice were treated with γ-secretase inhibitor dibenzazepine (DBZ, 4.28 mg/kg) or vehicle. Dormancy and reactivation were evaluated using micro-CT imaging (bone integrity), histomorphometric and immunofluorescence analysis (cytokeratin+ cell localization, distance from endosteal niche), qRT-PCR (Notch target genes *HRT1, HES1*), and H&E staining (liver micrometastases).	Human cell line (MDA-MB-231, luciferase- or turboGFP-transfected MDA-MB-231 and MCF-7), mouse cell line (4T1), xenograft mouse model (CD1 *nu/nu* mice) and human patient sample (Archived human primary breast cancers and bone metastases)	(112)
COCO (BMP inhibitor)	Overexpression	Breast cancer	Coco-induced dormancy was studied using *in vitro* and *in vivo* assays, including tail-vein metastasis models, EdU/Ki-67 staining, and PKH dye-retention to assess cell quiescence. Recombinant Coco/BMP4 treatments, Western blot, and qRT-PCR verified BMP-Smad pathway regulation, while sphere formation and bioluminescence imaging evaluated self-renewal and metastatic potential.	Murine cell lines (4TO7, 4T1, 66cl4, ErbB2-transformed mammary tumor cells isolated from MMTV-*Neu (YD)* mice), and mice model (syngeneic BALB/c mice, immunodeficient NOD/SCID/IL2Rγ–/– (NSG) mice)	(189)
PFKFB3	Overexpression	Breast cancer	Pfkfb3-driven dormancy escape was demonstrated using microarray-based gene expression profiling, qRT-PCR, and Western blotting to identify upregulation of Pfkfb3 in metastatic versus dormant cells. Functional validation involved 3D organoid growth assays, cell viability after 3PO inhibition, and *in vivo* tail-vein metastasis models in mice. Additionally, mammosphere formation, ELDA stemness assays, and flow cytometry (CD49f/CD24 markers) confirmed Pfkfb3’s role in dormancy exit and cancer stemness.	Murine cell line (D2.HAN, 4T1 series) and syngeneic mouse model (female BALB/c)	(190)

Modulating the dormancy-supportive microenvironment to overcome its protective role may restore chemosensitivity in dormant cancer cells. For instance, the endosteal niche helps sustain dormancy through cell-cell signaling involving the AXL tyrosine kinase, which is highly expressed by dormant myeloma cells. In a study of syngeneic mouse model, AXL inhibition (cabozantinib/BMS-777607) triggered the awakening of dormant DiD^hi^ 5TGM1-eGFP myeloma cells, as shown by flow cytometric loss of the dye 1,1’-dioctadecyl-3,3,3′,3′-tetramethylindodicarbocyanine (DiD) label, making them more responsive to chemotherapy (187). CAFs have been shown to influence OSCC dormancy by regulating DEC2 expression in a xenograft mouse model. Overexpression of DEC2 in OSCC cells induced dormancy and resistance to cisplatin, while CAF-derived CXCL1 downregulated DEC2, confirmed by qRT-PCR results, leading to the reactivation of dormant cells and contributing to tumor recurrence (188).

Reactivating dormant tumor cells into a proliferative phase followed by radio or chemotherapy presents a potentially effective approach to prevent tumor relapse. While numerous studies have proposed that awakening dormant cells could help overcome resistance to chemotherapy, translating this concept into clinical practice remains challenging. A significant challenge lies in ensuring every dormant cell reactivates and becomes susceptible to treatment. In fact, recent research suggests that such strategies might accelerate tumor recurrence and, in certain cases, worsen patient outcomes due to the persistence of residual quiescent cells.

### 6.3 Direct targeting of dormant cells

The key challenge with this approach lies in the fact that dormant cells are largely unresponsive to most conventional cytotoxic therapies, which are designed to target actively dividing cells. For example, pharmacological or genetic inhibition of autophagic pathways has been linked to decreased survival of dormant cells in mouse and human 3D *in vitro* and *in vivo* models of dormancy, while having minimal effect on cells that have transitioned to a proliferative state. Mechanistically, disrupting the autophagy in dormant breast cancer cells causes damaged mitochondria to accumulate and ROS levels to rise, ultimately triggering apoptosis (121). Various studies have confirmed that Bcl-2/Bcl-XL inhibitors, such as ABT-737 (191), ABT263 (192), Obatoclax combined with Tunicamycin (193), and A-1331852 (194), are effective in eliminating quiescent and senescent cells that express high levels of anti-apoptotic proteins. Similarly, DTPs have been found to be susceptible to ferroptosis, a form of programmed cell death triggered by the accumulation of lipid peroxides. The enzyme glutathione peroxidase 4 (GPX4) plays a protective role by preventing membrane lipid peroxidation, thereby inhibiting ferroptotic cell death (195). Inhibition of GPX4 with the compound RSL3 has been shown to selectively reduce the population of persistent cancer cells in various cancer cell lines, including breast (BT474), melanoma (A375), ovarian (Kuramochi), and lung (PC9), and as well as in A375 melanoma xenograft models (196). Furthermore, dormant medulloblastoma cells were effectively eradicated by the antineoplastic drug mithramycin following phenotypic screening (197). Similarly, in a model of pancreatic ductal adenocarcinoma, the IGF-1R tyrosine kinase inhibitor linsitinib was employed to target and eliminate residual cancer cells that had persistently survived despite the ablation of key oncogenes such as *c-MYC* or *K-RAS* (198). Moreover, Src kinase and ERK1/2 activities plays key role in maintaining dormancy and driving the transition to proliferation of dormant breast cancer cells. An *in vitro* and *in vivo* study shown that inhibition of Src kinase alone, using the Src inhibitor AZD0530, successfully prevented proliferation of dormant cells, but it did not induce cell death. However, combining AZD0530 (Src inhibitor) with AZD6244 (MEK1/2 inhibitor), triggered apoptosis in these dormant cells. This indicates that to effectively eliminate dormant DTCs, it is crucial to simultaneously target both survival pathways (such as Src signaling) and escape mechanisms (like ERK1/2 and MEK1/2) (185, 199).

The processes of inducing cellular dormancy and slowing cell proliferation are highly complex and may involve significant epigenetic reprogramming, yet the epigenetic landscape of slow-cycling cells remains largely unclear. Epigenetic therapies are being developed to target dormant cancer cells by suppressing key epigenetic enzymes involved in cellular dormancy and survival. One key regulator in this context is the epigenetic enzyme TET2, which has emerged as a critical factor in the survival and proliferation of slow-cycling cancer cells. Notably, TET2-mediated generation of 5-hydroxymethylcytosine (5hmC) has been associated with relapse risk and patient survival, indicating its potential as a therapeutic target for the eradication of slow-proliferating cells (200). In addition, Enzymes like histone deacetylases (HDACs) and lysine demethylases (KDMs), which play a crucial role in the survival of DTPs are being explored as potential targets. A key factor in this process is histone 3 (H3) demethylation at lysine 4 (H3K4), catalyzed by the KDM5 family. Several KDM inhibitors, such as GSK-J4, target enzymes like KDM2B (201), KDM5 (202), and KDM6A/B (203), and have shown significant potential in eliminating DTCs, particularly in lung and glioblastoma models. Their effectiveness is heightened when combined with taxane-platinum based chemotherapy and dasatinib (204, 205). Additionally, CPI-455, a highly specific KDM5 inhibitor, works by increasing H3K4 trimethylation (H3K4me3), which in turn reduces the population of DTP cells in cancer lines treated with chemotherapy. CPI-455 has shown promising results in preclinical studies involving lung, colon, melanoma, and breast cancers (206), making it a potential strategy for targeting dormant cancer cells.

The strategies focused on targeting senescent and/or quiescent cells, summarized in **Table 5**, must exhibit high efficacy to achieve complete elimination. However, achieving such efficiency could be challenging, as existing evidence indicates that if dormant cells persist or go untreated, they can become more aggressive, potentially leading to worse clinical outcomes (8). A number of therapeutic agents have been explored for their ability to either maintain or eradicate dormant cells/DTCs. **Table 6** summarizes clinically available and experimental drugs that modulate tumor dormancy/DTCs, their outcome, and current clinical trial status.

**Table 5:** Gene/pathways responsible for the direct killing of dormant cells.

Target gene/signaling pathway	Mechanism of target	Cancer model	Experimental methodology	Sample source	References
Bcl-2/Bcl-XL	Inhibition (ABT-737)	Lung cancer	ABT-737–induced dormancy disruption was investigated through cell viability (MTT) and soft agar colony assays to assess survival and self-renewal of liver cancer stem cells (LCSCs). PKH26 dye retention and FACS distinguished quiescent (PKH^high^) from proliferative (PKH^low^) cells, revealing selective killing of dormant LCSCs. JC-1 staining, cytochrome c/AIF immunofluorescence, and caspase 3/7 assays confirmed mitochondrial apoptosis. In xenograft models (NOD/SCID mice), tumor regression and reduced ALDEFLUOR^+^ stem cell content validated *in vivo* targeting of dormant CSCs by ABT-737.	Human patient tumor sample (LCSCs), and xenograft mouse model (NOD.Cg-Prkdcscid Il2rgtm1Wjl/SzJ (NSG) mice)	(191)
	PROTAC 753B - induced ubiquitination/degradation of BCL-XL and BCL-2; compared to DT2216, navitoclax (ABT-263), venetoclax (ABT-199), S63845, and Q-VD-OPH	Leukemia	753B treatment reduced senescence markers and SASP cytokine expression (IL-6, IL-8, IL-1β, CCR5), indicating clearance senescent cells. Apoptosis induction was verified by Annexin-V/DAPI staining and cleaved caspase-3/PARP western blotting. Increased BCL-XL expression in senescent cells and its degradation by 753B confirmed senolytic elimination of dormant leukemia cells, validated *in vitro* and *in vivo*.	Cell lines (AML, T-ALL, MPN-AML), Primary AML patient samples, patient-derived xenograft mouse model (NSG)	(207)
GPX4	Inhibition (RSL3)	Breast, melanoma, lung and ovarian cancer	Dormant DTP cells from lapatinib-treated cultures were exposed to GPX4 inhibitors. Ferroptotic death was confirmed through lipid ROS staining, rescue with ferrostatin-1 or DFO, and ineffectiveness of caspase inhibitor Z-VAD-FMK, establishing GPX4 inhibition-induced ferroptosis as the killing mechanism.	Human cell lines (BT474, PC9, Kuramochi, A375, MCF10A) and A375 xenograft mouse model (female athymic mice)	(196)
Sox2+ Cells	Inhibition (Mithramycin)	Sonic hedgehog subgroup medulloblastoma	Mithramycin based inhibition of SOX2^+^ cells were assessed using Alamar Blue viability assay, *in vitro* limiting dilution assays for self-renewal, secondary sphere formation tests, and *in vivo* xenograft growth suppression.	Mice model (Ptch1+/−, Sox2creER, Sox2-eGFP mice, were crossed to CD1 Patched1+/− mice. B6;129S6-Gt(ROSA)26Sortm9(CAG-tdTomato)Hze/J (Rosa-CAG-LSL-tdTomato) and 5- to 7-week-old NOD.Gc-Prkdcscid Il2rgtm1Wjl/SzJ (NSG) mice) and human patient sample	(197)
IGF-1R	Inhibition (Linsitinib)	Pancreatic cancer	Linsitinib-mediated IGF-1R inhibition was confirmed by immunoblot analysis of IGF-1R, AKT, and cleaved PARP in residual cancer cells, while apoptosis was quantified by flow cytometry of GFP-labeled cells; delayed tumor recurrence upon KRAS reactivation validated dormancy eradication *in vivo*.	Human cell lines (AsPC-1, MIA PaCa-2), primary mouse cell line (GFP-positive), mice model (CAG-LSL-GFP reporter strain, Pdx1-Cre transgenic mice, Cdkn2a knockout strain, TetO-H2B/GFP transgenic mice - orthotopic transplantation of tissues from transgenic mice to athymic nude mice)	(198)
Src and MEK1/2	Inhibition (AZD0530 + AZD6244)	Breast cancer	Dormant D2.0R breast cancer cells cultured on BME + COL1 were treated with AZD0530 (SFK inhibitor) and AZD6244 (MEK1/2 inhibitor). Apoptosis and viability were assessed by caspase-3/7 assays, Annexin V/PI staining, and MTS assays, while pERK1/2 levels were confirmed by immunoblotting. *In vivo* efficacy was validated in lung fibrosis mouse models via tail-vein injection and metastatic burden analysis.	Murine cell line (D2.0R, D2A1, 4T1, K7M2), human cell line (MDA-MB-231) and syngeneic mouse model (BALB/c)	(199)
KDM2B	Inhibition (GSK-J4)	Primary patient-derived Glioblastoma	Cell viability (MTT assay) and apoptosis (cleaved PARP, cleaved caspase-3, p21CIP1/WAF1 by Western blot) were measured. H3K36me2 accumulation confirmed KDM2B inhibition, while SOX2 and EZH2 reduction indicated loss of stemness. γH2AX immunostaining assessed DNA double-strand breaks, and sphere formation assays evaluated decreased GSC self-renewal.	Human cell line (About 4121, 4302, IN84), patient-derived primary glioblastoma cells (T115, 1587, T131, T133, T140, T143, T91, 017), and normal human astrocytes	(201)
KDM5	Inhibition (CPI-455)	Lung, Colon, Melanoma, and Breast Cancer	KDM5 inhibition was confirmed by increased H3K4me3 levels using Western blot and histone MS. Live-cell imaging tracked dose-dependent loss of DTP cells, while ALDH activity assays verified depletion of dormant, drug-tolerant subpopulations.	Human cell line (PC9, Colo205 SKBR3, Hs888, M14, SKBR3, EVSA-T)	(206)
ROCK	Inhibition (Y-27632)	Breast cancer	Dormant cancer cells were cultured under serum-free conditions to induce dormancy and treated with ROCK inhibitor Y-27632 at various time points (early, continuous, and late phases). Cell survival assays measured viability, while immunofluorescence staining for fibronectin visualized matrix assembly. Inhibition of ROCK disrupted fibronectin assembly and cell adhesion, leading to loss of dormant cell survival.	Human cell lines (BT549, BT474, HCC 1395, AU565, HCC 1419, HCC 1428, HCC 1806, HCC 1954, HCC 38, HCC 70, Hs578T, HCC 202, MCF7, MDA-MB-231, MDA-MB-231 BoM, MDA-MB-231 BrM2a, MDA-MB-231 LM2, MDA-MB-468, ZR-75-1)	(208)
YAP	Inhibition (XAV939)	Lung cancer	EGFR-mutant NSCLC cells were treated with OT (osimertinib + trametinib) + XAV939 for 3 weeks. After drug washout, cell regrowth was monitored to assess dormancy. YAP activity was tracked using a fluorescent YAP reporter and nuclear localization assays. CRISPR/Cas9-mediated YAP1 knockout and CellTiter-Glo/apoptosis assays confirmed that XAV939 blocked YAP activation, increased apoptosis, and prevented dormant cell survival and regrowth.	Human cell lines (PC9, HCC827, HCC4006, HCC2279, H1975, H3122, EBC-1), xenograft mice model (Female NCr nude, female NSG mice), and patient derived cell line (DFCI243)	(209)
TEAD	Inhibition (MYF-01-037)		MYF-01–37 was developed via structure-based docking to target the TEAD palmitoylation pocket. Split luciferase, qPCR, and Western blot assays confirmed inhibition of YAP-TEAD interaction and suppression of YAP target genes. Cell viability and apoptosis assays showed that MYF-01-37, combined with EGFR/MEK inhibitors, enhanced apoptosis and reduced dormant cancer cell survival.		

**Table 6:** Clinically available agents modulating dormancy or eliminating DTCs.

Drug/agent	Drug class/mechanism	Target cancer type	Clinical phase	Clinical trial ID	References
Palbociclib	CDK4/6 inhibitor	Breast cancer (ER+/HER2-)	FDA approved	PALOMA-1/2/3, NCT04841148	(168) (210) (211)
Zoledronic Acid (Zoledronate)	Bisphosphonate (bone resorption inhibitor)	Breast cancer (early stage with DTCs)	FDA approved	NCT0017206, NCT01545648	(212)
Tamoxifen	Selective estrogen receptor modulator (SERM)	Breast cancer (ER+)	FDA approved	Multiple trials	(213) (184)
Hydroxychloroquine	Autophagy inhibitor	Breast cancer (with DTCs)	Phase II	NCT04841148 (PALAVY), NCT03032406 (CLEVER), NCT04523857 (ABBY)	(214) (215)
Avelumab	Anti-PD-L1 immune checkpoint inhibitor	Breast cancer (with DTCs)	Phase II/III	NCT04841148 (PALAVY), NCT02926196 (A-Brave)	(216)
CB-839 (Telaglenastat)	Glutaminase inhibitor	Advanced solid tumors	Phase I/II	NCT02071862, NCT02771626	(217)
Entinostat	Histone deacetylase (HDAC) inhibitor	Breast cancer	Phase III	NCT02115282	(218, 219)
Azacitidine + Entinostat	DNA methyltransferase inhibitor + HDAC inhibitor	Advanced breast cancer (TNBC, hormone-resistant)	Phase II	NCT01349959	(220)
Saracatinib (AZD0530)	Src kinase inhibitor	Advanced breast cancer	Phase II	NCT00559507, ISRCTN23804370	(221, 222)
Navitoclax (ABT-263)	BCL-2/BCL-XL inhibitor	Lymphoid tumors	Phase I/II	NCT00406809	(223)
HC-5404	PERK pathway inhibitor	RCC, solid tumors with dormant metastasis	Phase I	NCT04834778	(224)
Everolimus	mTOR inhibitor	Breast cancer (ER+ with DTCs)	Phase II	NCT03032406 (CLEVER)	(215)
Lenalidomide	Immunomodulatory drug (IMiD)	Multiple myeloma	FDA approved	NCT03710603	(225)
Pomalidomide	Immunomodulatory drug (IMiD)	Multiple myeloma	FDA approved	NCT02188368, NCT01311687, MM-014 trial	(226, 227)
Ribociclib	CDK4/6 inhibitor	Breast cancer (HR+/HER2-)	FDA approved	NCT01958021 (MONALEESA-2)	(228)
Abemaciclib	CDK4/6 inhibitor	Metastatic solid tumors, Breast cancer (HR+/HER2-, with DTCs)	FDA approved	NCT04594005, NCT03155997 (MonarchE)	(229–231)
Venetoclax	BCL-2 inhibitor	CLL, AML, lymphoid malignancies	Phase III/FDA approved	NCT02993523(VIALE-A)	(232)
Ibrutinib	BTK inhibitor	CLL, MCL, Waldenström macroglobulinemia	FDA approved	NCT01724346, NCT01722487, NCT01611090	(233)
Trametinib	MEK1/2 inhibitor	Melanoma	FDA approved	NCT01245062	(234)

## 7 Challenges and future perspectives

Tumor dormancy plays a critical role in cancer progression, offering a unique therapeutic window for potential interventions. Understanding the mechanisms that keep tumor cells dormant and identifying the triggers for their reactivation are among the central challenges in cancer biology today. Numerous signaling cascades and external factors are involved in this process that dynamically shift the cancer cells between dormancy and proliferation. This complexity suggests that straightforward solutions are unlikely. However, mechanistic insights into dormancy have laid the groundwork for therapeutic strategies aimed at targeting this stable phase of cancer development to prevent recurrence and improve treatment outcomes.

Although this review discusses the mechanistic role of hypoxia in inducing and maintaining tumor dormancy, translating these insights into clinical applications remains a major challenge. The hypoxic TME is highly dynamic and spatially heterogeneous, leading to variable responses among different tumor types and even within the same lesion. Moreover, the dual role of hypoxia in both enforcing dormancy and facilitating reactivation/angiogenesis complicates the design of targeted interventions. To address these challenges, bioengineered models inspired by tissue engineering are increasingly being utilized to recapitulate critical aspects of the tumor microenvironment. These include three-dimensional (3D) biomaterial-based cultures, microfluidic platforms, and organ-on-chip systems that enable the controlled study of hypoxia-driven dormancy mechanisms in physiologically relevant settings. They incorporate key microenvironmental elements such as ECM, niche cells, or combinations of both, to better understand how the microenvironment influences the regulation of tumor dormancy (235). However, experimental models used to investigate tumor dormancy - whether *in vitro*, *in vivo* or *ex vivo*, continue to face obstacles in reproducing the intricate dynamics of tumor niches seen in patients. Developing models that more accurately simulate real-time behaviors within the TME (236), potentially through bioengineering breakthroughs, combined with *in silico* models (including those that involve the latest AI/ML-based models) to predict cellular behaviors in various contexts, will be essential for generating reliable research data. This approach requires iterative strategies, wherein the advancements in higher order *in vitro* tumor dormancy-related models is coupled to the development and/or refinement of the AI/ML-related algorithms. Both these approaches may most plausibly lead to the development of models that can better recapitulate the cellular and molecular features of tumor dormancy, that can improve our mechanistic understanding of this pathological phenomenon as well as enable their deployment for anti-cancer drug testing.

Targeting cells during the window of cancer dormancy requires improved diagnostic tools capable of detecting latent disease before resistant macroscopic lesions emerge. Liquid biopsy presents a potential strategy for identifying dormant cancer cells. These blood-based tests offer a minimally invasive alternative to traditional tumor biopsies that enables molecular analysis of circulating tumor DNA (ctDNA), tumor-derived extracellular vesicles, and CTCs. The U.S. Food and Drug Administration (FDA) has already approved three liquid biopsy tests, including the cobas^®^ EGFR Mutation Test v2, Guardant360^®^ CDx, and FoundationOne^®^ Liquid CDx, which target ctDNA. Additionally, FDA-approved devices like CellSearch^®^, Parsortix^®^, ClearCell^®^ FX1, and Vortex VTX-1 are used for isolating CTCs (237). While these developments are promising, translating such insights into clinical practice and increasing accuracy remains in its early stages, although significant progress is expected in the near future.

Nevertheless, a deeper understanding of how DTCs transition between quiescence/senescence, persistence, and reactivation remains a central challenge. Many questions remain unanswered. It is unclear whether the mechanisms governing tumor dormancy, act independently or represent interconnected stages of tumor progression. Additionally, different cancer types may employ distinct dormancy programs shaped by their oncogenic and microenvironmental contexts. Future investigations should integrate single-cell multi-omics, lineage tracing, and spatial transcriptomics to trace molecular signatures distinguishing dormant from proliferative states across diverse metastatic niches. Particular attention should be given to the role of epigenetic plasticity, metabolic rewiring, and immune surveillance in maintaining dormancy. Translating dormancy biology into clinical benefit remains complex. Dormant cell behavior is profoundly shaped by the heterogeneous tumor microenvironment and by patient-specific variables, including immune composition, stromal architecture, and systemic metabolism. The transient and often undetectable nature of dormant cells further complicates longitudinal monitoring and therapeutic evaluation. Bridging this translational gap will require collaborative efforts that integrate molecular biologists, computational scientists, and clinicians. Advances in liquid biopsy-based detection, real-time imaging, and data-driven predictive models hold promise for tracking dormant cell dynamics and for designing strategies that either safely maintain dormancy, strategically reawaken and eradicate dormant cells, or directly eliminate residual disease before relapse occurs.

## 8 Conclusion

In conclusion, this article provides a thorough exploration of the major signaling pathways, as well as the internal and external factors, that either keep DTCs in a dormant (quiescent and/or senescent) state or trigger their reactivation, leading to metastasis. Many of these mechanisms are specific to certain organs, highlighting the strong connection between dormancy and the surrounding tissue micro-environment. Because of this, targeting DTCs will likely require a highly personalized approach. Since DTCs can remain dormant for long periods, they present a unique window for therapeutic intervention. Furthermore, this review is intended to guide future molecular characterization studies aimed at identifying the key molecular determinants that distinguish DTCs from embryonic and adult stem cells, as well those that exhibit cancer stem cell-like features.
